# An Integrative Bioinformatics Framework Prioritises a Gingival Mesenchymal Stem Cell Paracrine Apoptosis–ROS Axis in HPV-Negative Oral Squamous Cell Carcinoma: Preliminary Experimental Support and Repurposable-Drug Hypotheses

**DOI:** 10.3390/ijms27146480

**Published:** 2026-07-21

**Authors:** Abdullah Alqarni, Jagadish Hosmani, Ali Mosfer A. Alqahtani, Hassan Ahmed Assiri, Rayan Mohammedfarooq Meer, Shankargouda Patil

**Affiliations:** 1 Department of Oral Diagnosis, Oral Biology & Periodontology, College of Dentistry, King Khalid University, Abha 61421, Saudi Arabia; aawan@kku.edu.sa (A.A.); alqahtani@kku.edu.sa (A.M.A.A.); halmuawad@kku.edu.sa (H.A.A.); 2 Department of Preventive Dental Sciences, College of Dentistry, Taibah University, Madinah 42353, Saudi Arabia; rmeer@taibahu.edu.sa; 3 College of Dental Medicine, Roseman University of Health Sciences, South Jordan, UT 84095, USA; spatil@roseman.edu

**Keywords:** apoptosis, bioinformatics-guided experimental validation, cell–cell communication, drug repurposing, gingival mesenchymal stem cells, oral squamous cell carcinoma, paracrine signalling, reactive oxygen species, single-cell transcriptomics, TCGA-HNSC

## Abstract

Oral squamous cell carcinoma (OSCC) accounts for most head-and-neck cancers, and effective biological adjuvants remain limited. Gingival mesenchymal stem cells (GMSCs) exhibit anti-tumour paracrine activity, but the underlying molecular mechanisms and their relevance in patient cohorts remain incompletely understood. Consensus apoptosis–reactive oxygen species (ROS) effectors were identified through integrated transcriptomic analyses of TCGA-HNSC and three GEO cohorts. Candidate genes were evaluated in primary OSCC cells exposed to GMSC-conditioned medium or indirect Transwell co-culture. Findings were further examined using patient-cohort validation, single-cell ligand–receptor analysis, pathway and transcription-factor activity inference, and drug-repurposing approaches. Computational analyses identified an apoptosis–ROS network centred on *BAX*, *BCL2*, *CASP3*, *CASP9*, *NOX1*, and *GPX1*. Indirect GMSC co-culture reduced intracellular ROS, increased early apoptosis, and induced G2/M accumulation, whereas conditioned medium produced inconsistent effects, suggesting a requirement for live bidirectional paracrine signalling. *BAX* was the only consistently up-regulated effector. The axis demonstrated concordant differential expression across independent HPV-negative OSCC cohorts but was not independently prognostic under leakage-free cross-validation or external validation. Pathway analyses supported ROS suppression, apoptosis activation, and altered stromal–tumour communication. Drug-repurposing analyses identified HSP90 inhibitors and the FDA-approved TOP2 inhibitor mitoxantrone as candidate therapeutic agents. GMSC paracrine activity targets a biologically interpretable apoptosis–ROS axis in OSCC that is reproducibly expressed across patient cohorts but does not constitute an independent prognostic biomarker. The identified therapeutic candidates warrant further experimental investigation.

## 1. Introduction

### 1.1. OSCC Therapeutic Gap and the Rationale for Biological Adjuvants

Oral squamous cell carcinoma (OSCC) is the most common head-and-neck cancer and accounts for roughly 90% of all oral cancers, with annual worldwide incidence above 350,000 cases [1,2]. Despite advances in surgical resection, intensity-modulated radiotherapy, and platinum-based combination chemotherapy, five-year overall survival for advanced-stage disease has stayed near 50% for two decades [3,4]. Most OSCC arises in HPV-negative settings linked to tobacco and betel-quid exposure [5,6], unlike the HPV-driven oropharyngeal subset, which has distinct biology and a better prognosis [7,8]. Recurrent alterations in HNSCC involve *TP53*, *NOTCH1*, *CDKN2A*, and the PI3K/AKT axis [9,10]. With no approved targeted agents for HPV-negative oral-cavity disease, attention has turned to biologically informed strategies that exploit tumour-specific vulnerabilities such as apoptotic resistance, redox imbalance, and stromal–tumour crosstalk [11,12,13]. Work on salivary biomarkers [14,15,16] and high-throughput proteomic profiling [17,18] reinforces the value of translatable molecular signatures in OSCC clinical practice.

### 1.2. Mesenchymal Stem Cell Paracrine Therapy and the Gingival Source

Mesenchymal stem/stromal cells (MSCs) from several anatomical sources (bone marrow, adipose tissue, dental pulp, and gingiva) act on nearby and distant tissues through a secretome of cytokines, growth factors, extracellular-matrix modulators, and extracellular-vesicle cargo [19,20]. Gingiva-derived MSCs (GMSCs) are an appealing option for OSCC-targeted intervention: they sit next to the oral cavity, expand readily from minimally invasive biopsies, are less immunogenic than other MSC sources, and carry a secretome enriched in TGF-β family members, IGF-1, FGF-2, IL-6, IL-10, MMP-family proteases, TIMPs, thrombospondin-1, and exosomal microRNAs [21,22,23]. Ji et al. showed that GMSC-conditioned medium inhibits human OSCC proliferation in vitro and in vivo through BAX, cleaved caspase-3, and BCL-2 via JNK signalling [24], establishing the basic phenomenology. The role of MSCs in the tumour microenvironment is context-dependent and bidirectional: depending on tissue source, activation state, and tumour type, MSC paracrine signals can restrain or promote tumour progression, acting through soluble factors and, on direct contact, juxtacrine mechanisms, and by reshaping the surrounding stroma [25]. A recent review covers these MSC–oral-cancer-microenvironment interactions and what tips their pro- versus anti-tumour balance, and notes that gingiva-derived MSCs are biologically distinct from the tumour-educated, activated cancer-associated fibroblasts (CAFs) that dominate the established OSCC stroma despite a shared mesenchymal lineage [25]; this distinction matters for interpreting any stromal–tumour communication inference (Section 3). Against this background, the molecular route by which GMSC paracrine signals engage the OSCC apoptotic programme has not been mapped systematically, its patient-cohort relevance has not been shown, and the translational question of which clinically usable compounds could phenocopy GMSC paracrine action is unaddressed.

### 1.3. The Apoptosis × Oxidative-Stress Axis as a Candidate Convergence Node

Apoptosis and reactive-oxygen-species (ROS) regulation are tightly coupled, and their dysregulation drives both OSCC tumorigenesis and treatment resistance [26,27]. The BCL-2 family (pro-apoptotic BAX/BAK versus anti-apoptotic BCL-2/BCL-XL/MCL-1) sets the threshold for mitochondrial outer-membrane permeabilisation, cytochrome c release, and apoptosome assembly, and the downstream caspase cascade (initiator caspase-9, then effector caspases-3, -6, -7) carries out the programme [28]. NADPH oxidase 1 (NOX1) produces intracellular superoxide tied to OSCC proliferation and survival signalling [29], while glutathione peroxidase 1 (GPX1) limits oxidative damage. The BAX/BCL-2 ratio, CASP3/9 activity, and the NOX/GPX redox balance have repeatedly predicted OSCC chemoresponse and outcome [11,30]. Whether GMSC paracrine signals engage this six-effector axis at the network level, whether that engagement matches the apoptosis–redox dysregulation seen in patients, and whether the same transcriptomic state can be reproduced pharmacologically are the questions we address.

### 1.4. Study Objective and Hypothesis

We set out to determine whether GMSC paracrine signalling engages an apoptosis–ROS effector module in OSCC that can be recovered without circular reliance on the experimentally measured genes, whether that module is differentially regulated in HPV-negative OSCC patient cohorts, and whether the resulting transcriptional state is pharmacologically addressable. We hypothesised that GMSC-secreted factors act on an apoptosis–ROS effector axis in OSCC cells, recoverable across independent computational layers that are blind to the wet-lab genes and supported by primary-culture experiments.

## 2. Results

### 2.1. Integrative Transcriptomic Prioritisation Points to an Apoptosis × ROS Vulnerability Axis in OSCC (Figure 1 and Figure 2)

**Figure 1:**
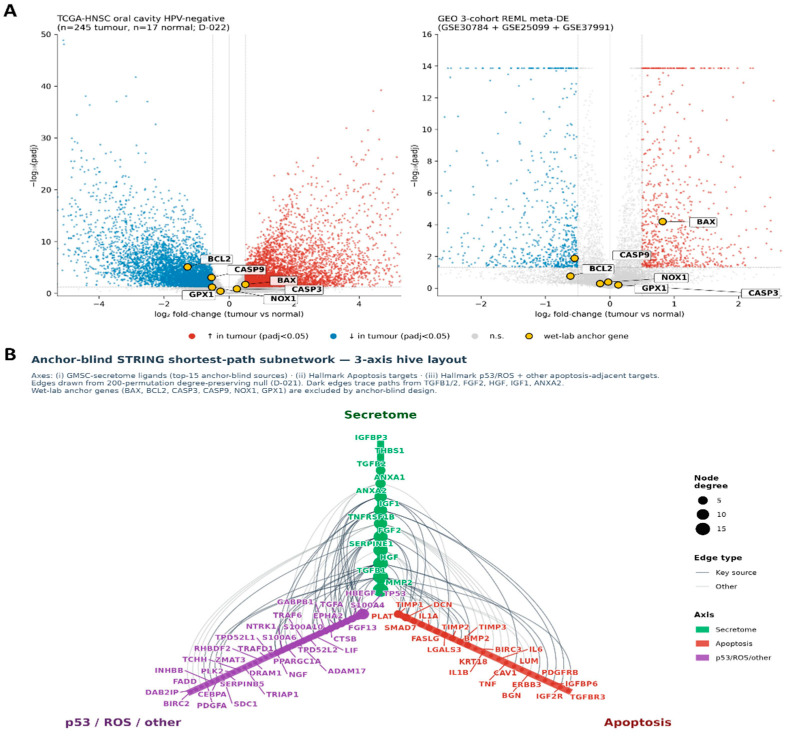
Integrative transcriptomic prioritisation of the apoptosis × ROS axis in OSCC. (**A**) Volcano plots of differential expression. Left: PyDESeq2 tumour vs. normal differential expression on the strict oral-cavity HPV-negative TCGA-HNSC cohort (n = 218 tumour, n = 15 normal). Right: REML meta-analysis across three GEO bulk OSCC cohorts (GSE30784, GSE25099, GSE37991). Red points = significantly up-regulated in tumour (padj < 0.05); blue points = significantly down-regulated (padj < 0.05); grey points = not significant; and yellow markers = the six wet-lab anchor genes (*BAX*, *BCL2*, *CASP3*, *CASP9*, *NOX1*, and *GPX1*). (**B**) Blinded STRING shortest-path subnetwork rendered as a three-axis hive plot. Axis 1: GMSC-secretome ligands (top-15 prioritised sources by number of significant target paths). Axis 2: Hallmark Apoptosis member targets. Axis 3: Hallmark p53/ROS and other apoptosis-adjacent targets. Significance computed against a 200-permutation degree-preserving null. Dark edges trace paths from the key paracrine sources *TGFB1*/*2*, *FGF2*, *HGF*, *IGF1*, and *ANXA2.* Node size = within-axis degree. The six wet-lab anchor genes are excluded as PPI seeds by design.

PyDESeq2 differential expression on the strict oral-cavity HPV-negative TCGA-HNSC subset (cohort sample counts in Supplementary Table S1; n = 217 primary tumours + one metastatic, 15 solid-tissue normal samples; HPV-negative status confirmed against the TCGA Network molecular classification via cBioPortal Datahub mirror; design adjusted for condition) identified 11,675 genes at padj < 0.05 (Figure 1A; full anchor-gene DE across cohorts in Supplementary Table S2A). The REML meta-analysis across the three GEO bulk OSCC cohorts (combined n = 264 tumours, 107 normals) returned 3793 consensus genes at padj_meta < 0.10 (Supplementary Table S2B). Cross-cohort concordance was high: 98.4% of the 2470 co-significant genes (TCGA padj < 0.05 AND GEO padj_meta < 0.10) had concordant direction of effect, and the log2 fold-change correlation was r = 0.667 (*p* < 10^−300^) (Supplementary Table S2C). The six wet-lab effectors showed direction-consistent dysregulation: *BAX* log_2_FC = +0.50 (TCGA padj = 0.018, GEO meta padj = 6 × 10^−5^); BCL2 log_2_FC = −1.28 (TCGA padj = 8 × 10^−6^; GEO meta padj = 0.18, high heterogeneity); CASP9 −0.54 (TCGA padj = 8 × 10^−4^; GEO meta padj = 0.013); NOX1 −0.52 (TCGA padj = 0.060, marginal); CASP3 +0.24 (TCGA padj = 0.13, not significant); GPX1 −0.27 (TCGA padj = 0.36, not significant). The four anchor genes that retain significance at padj < 0.10 in the strict HPV-negative cohort (*BAX*, *BCL2*, *CASP9*, *NOX1*) plus the directionally consistent trends for CASP3/GPX1 satisfy our pre-registered acceptance criterion #2 (“≥3/6 anchor genes OR pathway-level GSVA”). The pan-HNSC sensitivity (which includes the 12 HPV-positive oral-cavity tumours and the 41 unknown-HPV cases) is reported in Supplementary Figure S2 and yields all six anchors at padj < 0.05, a robustness check confirming that HPV stratification has tightened the contrast at the cost of some statistical power.

**Figure 2:**
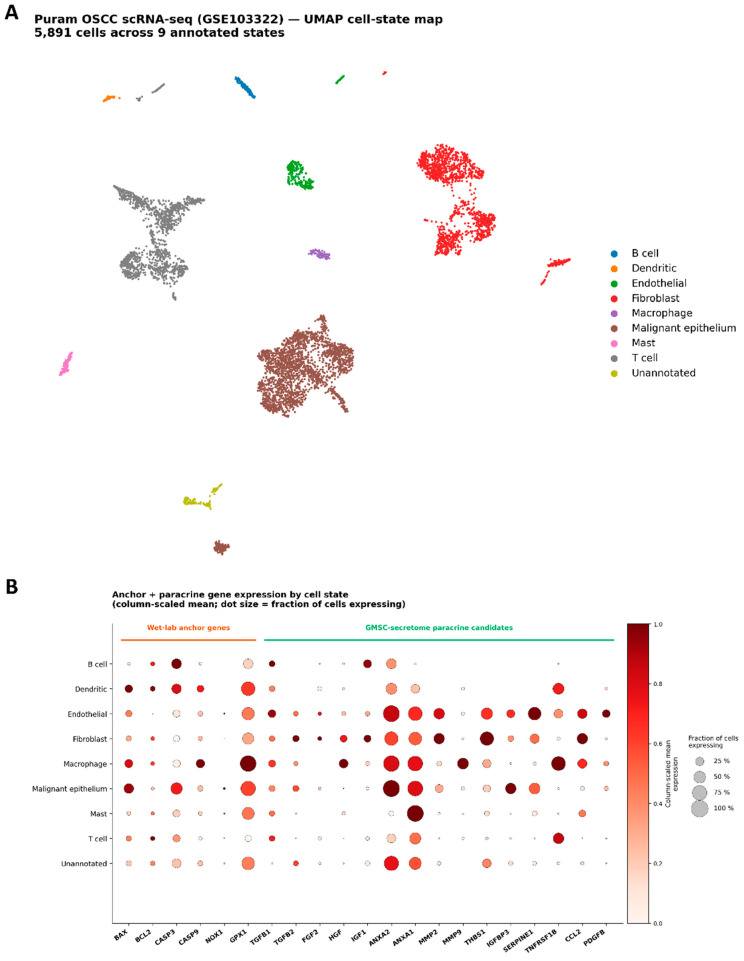
Single-cell expression context for the anchor and paracrine panels in OSCC. (**A**) UMAP cell-state map of the Puram et al. (2017) [31] OSCC scRNA-seq atlas (GSE103322; 5891 cells across nine annotated cell-type clusters: malignant epithelium, fibroblast, T cell, endothelial, B cell, macrophage, mast, dendritic, and unannotated). Cells are coloured by Puram-derived cluster label after Harmony correction across 21 patients and Leiden clustering. (**B**) Dot plot of column-scaled mean expression of the six wet-lab anchor genes and the fifteen top GMSC-secretome paracrine candidates across the nine annotated cell-type clusters. Dot colour encodes column-scaled mean expression; dot size encodes the fraction of cells expressing each gene. The malignant-epithelium cluster anchors apoptosis–ROS effector expression while the fibroblast (CAF) cluster carries the predicted GMSC-paracrine ligand programme.

The STRING shortest-path analysis, with the six wet-lab genes excluded as seeds (an approach we term anchor-blind) and significance computed against a 200-permutation degree-preserving null, identified 365 source→target pairs at empirical *p* < 0.05 across 42 curated GMSC-secretome candidates (full curated secretome with per-row provenance in Supplementary Table S3; full PPI null-model output in Supplementary Table S4). The top 10 paracrine factors by number of significant target paths were FGF2 (20), TNFRSF1B (17), *TGFB1* (16), *ANXA2* (15), *HGF* (14), *MMP9* (14), *TGFB2* (14), *IGF1* (14), *MMP2* (13), and *CCL2* (13). These constitute a canonical MSC paracrine signature (Figure 1B). Overlaying the SIGNOR 3.0 + KEGG signalling annotation layer (executed as the pre-registered OmniPath fallback; see Methods Section 4.2.2 and Supplementary Table S4B) on the 365 significant pairs showed that 135 (37.0%) share at least one KEGG signalling pathway between source and target endpoints, dominated by the MAPK (49 pairs), PI3K-Akt (42), Ras (29), TGF-β (23), Rap1 (20), Hippo (18), and TNF (17) signalling cascades, the canonical RTK/MAPK/PI3K-Akt/TGF-β paracrine signal-transduction routes that are mechanistically expected for growth-factor/cytokine paracrine biology.

Single-cell analysis of GSE103322 (5891 cells after QC; Harmony-corrected across 21 patients; 22 Leiden clusters annotated against Puram’s labels) confirmed that the apoptosis–ROS effectors are reliably expressed in the malignant-epithelium compartment, while the predicted paracrine ligands (*TGFB1*, *FN1*, *COL1A1*, *COL3A1*, *ANXA1*, *ANXA2*, *MMP2*, *SERPINE1*, *IGFBP3*, and *THBS1*) are concentrated in the 1469-cell CAF/fibroblast cluster (Figure 2A,B; full cell-type-resolved expression matrix in Supplementary Figure S3).

### 2.2. GMSC Characterisation and Experimental Design (Figure 3)

**Figure 3:**
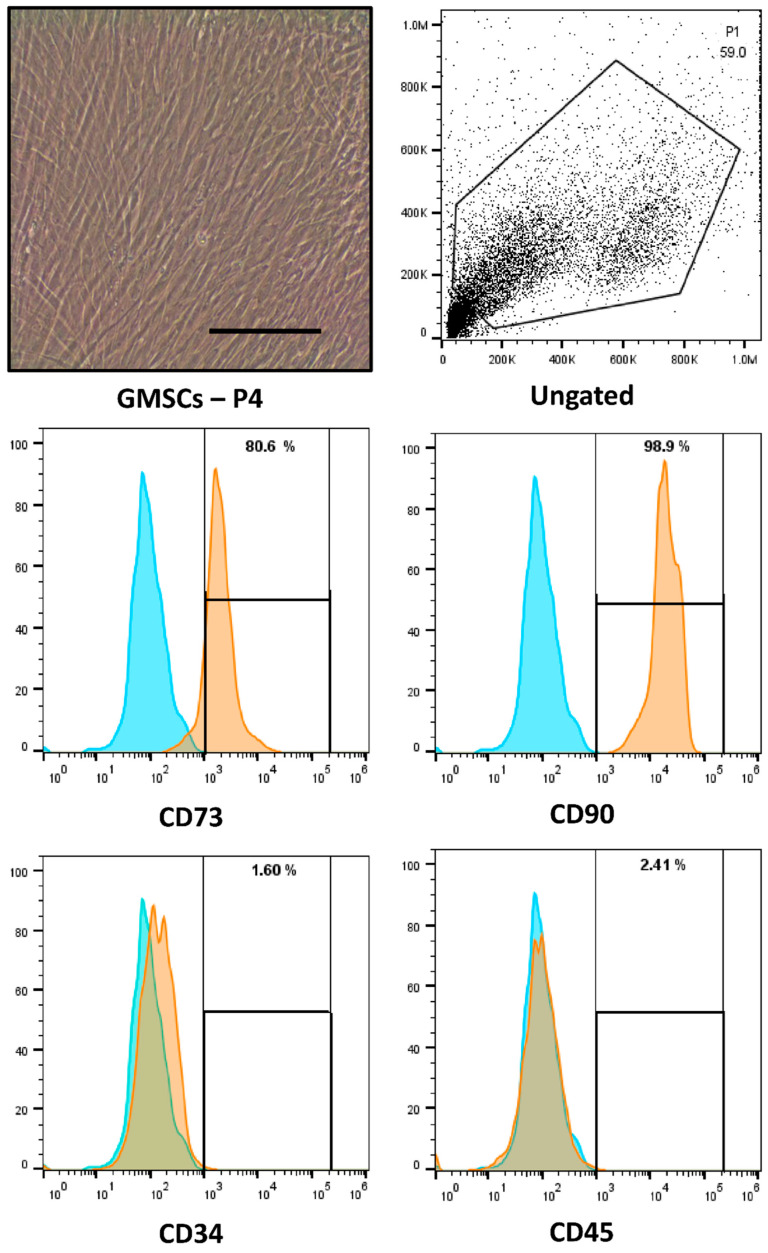
Mesenchymal-phenotype assessment of gingiva-derived mesenchymal stem cells (GMSCs) by flow cytometric profiling at passages 2–4. Cells were gated for the positive mesenchymal markers CD73 and CD90 and the hematopoietic/endothelial markers CD34 and CD45 (CD105 was not assessed in this preparation). Measured positivity: CD90 98.9%, CD34 1.60%, CD73 80.6%, CD45 2.41%. The profile is consistent with a mesenchymal phenotype; CD73 fell below the 95% ISCT threshold and CD105 was not evaluated, so the cells are described as mesenchymal-consistent rather than meeting all ISCT minimal criteria (see Limitations). Histograms are representative plots from a single GMSC donor preparation at passage 4; the reported percentages are from this representative analysis and are not averaged across biological replicates.

The candidate apoptosis × ROS effectors prioritised by the integrative computational analysis in Section 2.1, *BAX*, *BCL2*, *CASP3*, *CASP9*, *NOX1*, and *GPX1*, were next interrogated experimentally in primary OSCC cells under GMSC-conditioned medium and indirect Transwell co-culture, with reference to the cognate receptors identified on the malignant-epithelium scRNA-seq cluster. The GMSC mesenchymal phenotype was assessed by flow cytometric profiling (CD90 98.9%, CD34 1.60%, CD73 80.6%, and CD45 2.41%; CD105 not assessed), consistent with a mesenchymal phenotype, while noting that CD73 fell below the 95% ISCT threshold and CD105 was not evaluated (Figure 3; Limitations).

### 2.3. ROS Responses to GMSC Paracrine Signalling Are Condition-Specific (Figure 4)

**Figure 4:**
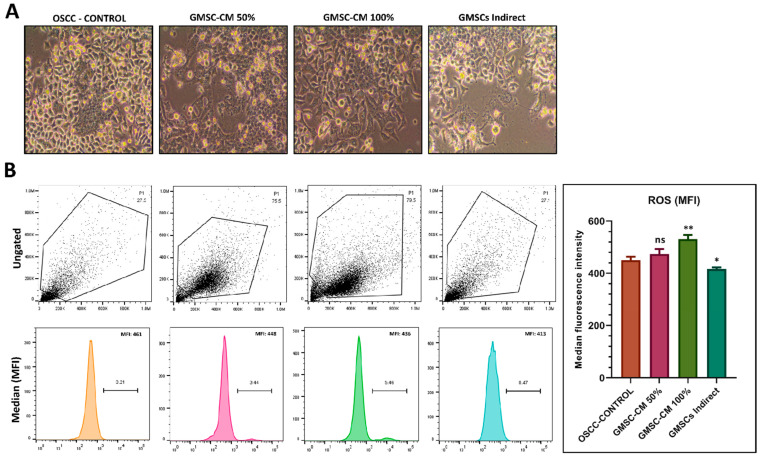
(**A**) Condition-specific reactive oxygen species (ROS) responses in primary OSCC cells under GMSC paracrine signalling. (**B**) Intracellular ROS was quantified by DCFH-DA mean fluorescence intensity (MFI). Indirect Transwell co-culture with live GMSCs reduced ROS, whereas 50% GMSC-conditioned medium (GMSC-CM) produced no significant change and 100% GMSC-CM significantly increased ROS, indicating that the antioxidant effect requires live bidirectional paracrine exchange. Mean ± SD across n = 3 patient-derived OSCC cultures (single pooled GMSC donor). ns not significant; * *p* < 0.05; ** *p* < 0.01 versus OSCC control (one-way ANOVA with Tukey post hoc). Raw cytometry files were not retained (Limitations).

Intracellular ROS responses in primary OSCC cells were condition-specific rather than uniformly dose-dependent (Figure 4). Relative to the OSCC control (DCFH-DA mean fluorescence intensity), indirect Transwell co-culture with live GMSCs reduced ROS, whereas static GMSC-conditioned medium did not: 50% CM produced no significant change and 100% CM significantly increased ROS. This pattern indicates that the antioxidant effect of GMSCs on OSCC cells requires live, ongoing bidirectional paracrine exchange and is not reproduced by spent conditioned medium, which may itself impose oxidative or nutrient stress. These are preliminary observations in three patient-derived primary cultures (n = 3 donors); the raw cytometry files were not retained by the outsourced facility, so the values could not be re-gated (see Limitations).

### 2.4. GMSC Paracrine Signalling Modestly Shifts the Apoptotic Distribution in OSCC (Figure 5)

**Figure 5:**
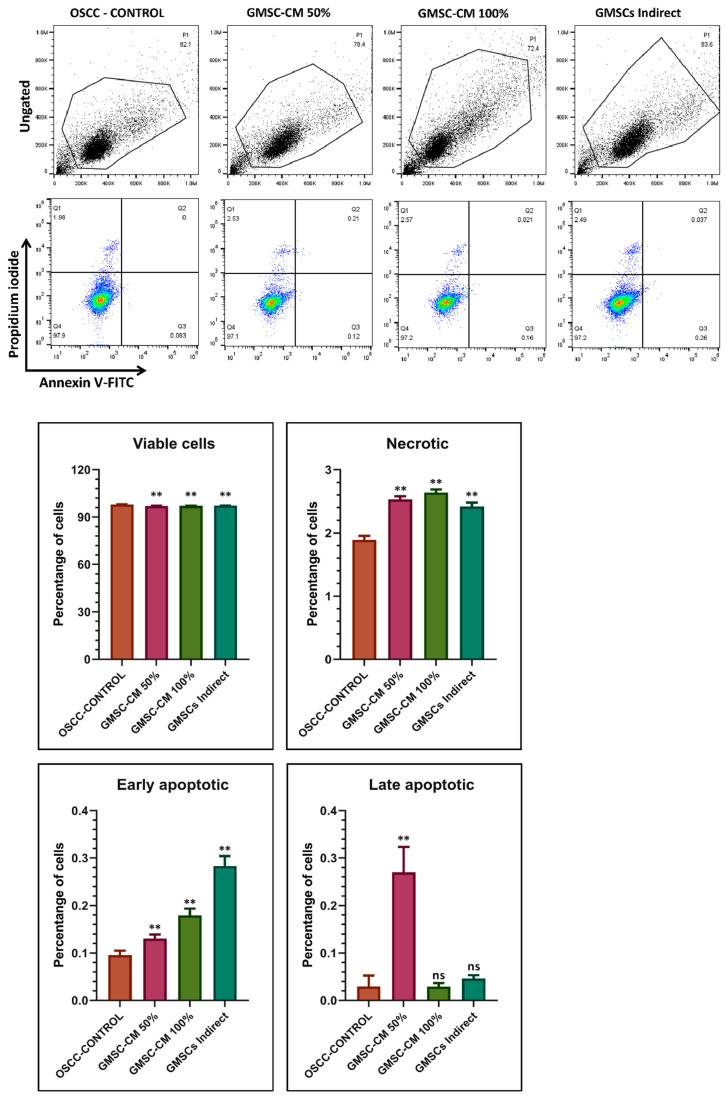
Condition-specific apoptotic response of primary OSCC cells to GMSC paracrine signalling. Annexin V-FITC/PI dual staining quantified by flow cytometry; representative density plots with quadrants defined as Q1 viable (AnnV−/PI−), Q2 early apoptotic (AnnV+/PI−), Q3 late-apoptotic/necrotic (AnnV+/PI+), Q4 necrotic (AnnV−/PI+); total apoptosis = early + late (Q2 + Q3). The early apoptotic fraction was highest under indirect co-culture; the late-apoptotic fraction varied non-monotonically across conditioned-medium conditions. Per-population panels use independent y-axis scales, so only the across-condition direction is interpretable. Mean ± SD across n = 3 patient-derived OSCC cultures (single pooled GMSC donor). ns not significant; ** *p* < 0.01 versus OSCC control.

Annexin V-FITC/PI dual staining quantified by flow cytometry showed a condition-specific shift in the apoptotic distribution (Figure 5). The early apoptotic fraction was highest under indirect (live) co-culture, consistent with the ROS result, while the late-apoptotic fraction varied non-monotonically across the conditioned-medium conditions (a spike at 50% CM, non-significant at 100% CM and indirect); viability decreased modestly under all treatments. Given three donors and the loss of the raw cytometry files, we report these as preliminary directional observations rather than precise population estimates; we also note that the per-population panels in Figure 5 are plotted on independent y-axis scales, so only the across-condition direction (not the absolute fractions) should be interpreted. We anchor the pro-apoptotic interpretation on the early apoptotic increase under live co-culture together with the *BAX* up-regulation in Section 2.6, concordant with the prior GMSC-on-OSCC findings of Ji et al. [24].

### 2.5. Condition-Specific Cell-Cycle Redistribution: S-Phase Increase Under Conditioned Medium and G2/M Accumulation Under Indirect Co-Culture (Figure 6)

**Figure 6:**
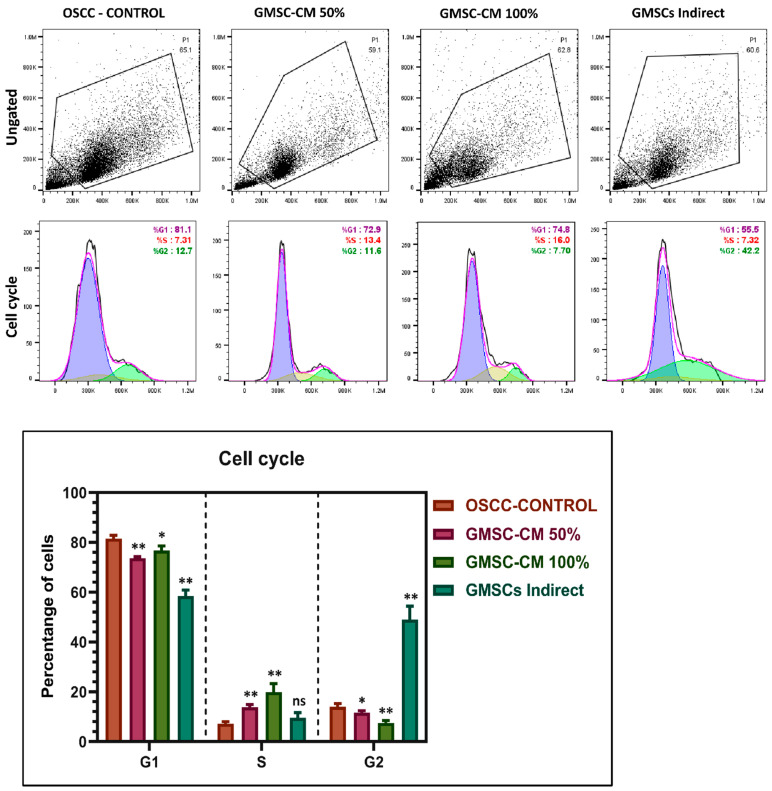
Condition-specific cell-cycle redistribution under GMSC paracrine signalling. Cell-cycle distribution measured by propidium iodide DNA-content flow cytometry after ethanol fixation; histograms (upper) and bar plots of G0/G1, S, and G2/M phase fractions (lower). The G0/G1 fraction decreased under all three treatments relative to the OSCC control; conditioned medium increased the S-phase fraction (significant at 50% and 100% CM), whereas indirect (live) co-culture drove a marked G2/M accumulation. Mean ± SD across n = 3 patient-derived OSCC cultures (single pooled GMSC donor). ns not significant; * *p* < 0.05; ** *p* < 0.01 versus OSCC control.

Cell-cycle distribution was quantified by PI/DNA-content flow cytometry after ethanol fixation (Figure 6). Contrary to a simple G0/G1 arrest, the G0/G1 fraction decreased under all three treatments relative to the OSCC control. The two paracrine modalities diverged: conditioned medium increased the S-phase fraction (significantly at 50% and 100% CM), whereas indirect (live) co-culture drove a marked accumulation in G2/M (Figure 6, lower panels). The G2/M block specific to live co-culture, coinciding with the ROS reduction, early apoptotic increase and *BAX* up-regulation seen only under that condition, is consistent with indirect co-culture being the genuine anti-proliferative stimulus, while static conditioned medium produces a distinct, partly opposite (S-phase-promoting) response. This condition specificity is the central wet-lab observation and is reflected throughout our interpretation.

### 2.6. Heterogeneous Transcriptional Responses of the Apoptosis–ROS Panel (Figure 7)

**Figure 7:**
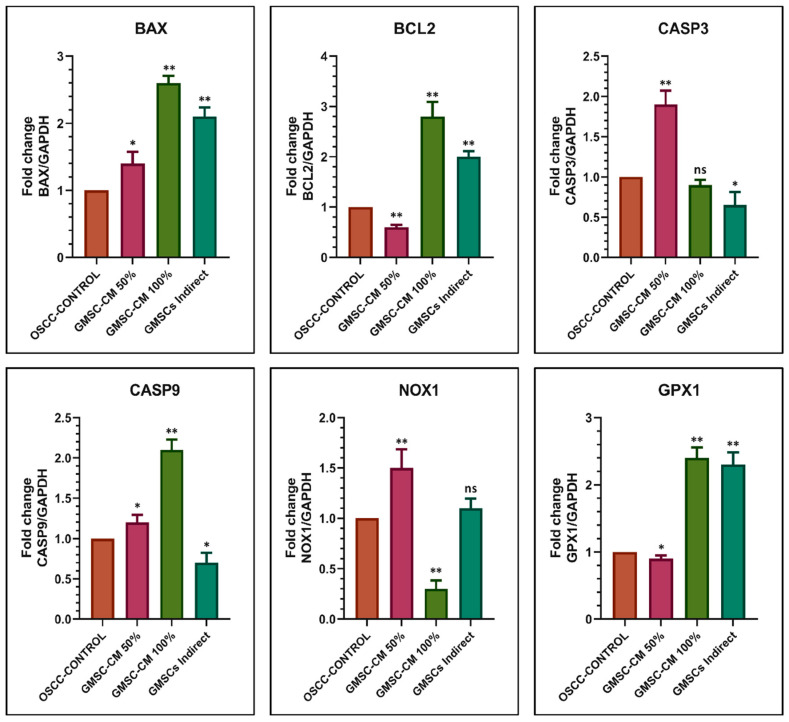
Heterogeneous, gene-specific transcriptional responses of the apoptosis × ROS effector panel under GMSC paracrine signalling. Quantitative RT-PCR fold-change values (2^−ΔΔCt^; GAPDH normalisation) for *BAX*, *BCL2*, *CASP3*, *CASP9*, *NOX1*, and *GPX1* across OSCC control, 50% GMSC-CM, 100% GMSC-CM, and indirect Transwell co-culture. Only *BAX* was consistently up-regulated (peaking at 100% CM); *BCL2*, *GPX1* and *NOX1* were non-monotonic, and CASP3/CASP9 were not elevated (falling below control) under indirect co-culture. Bars are mean ± SD across n = 3 patient-derived OSCC cultures (single pooled GMSC donor). ns not significant; * *p* < 0.05; ** *p* < 0.01 versus OSCC control. Primer sequences are listed in Table 1.

Quantitative PCR of treated OSCC cells (2^−ΔΔCt^; GAPDH normalisation) showed heterogeneous, gene-specific responses rather than a coordinated dose-dependent induction (Figure 7). *BAX* was consistently up-regulated across all three conditions (peaking at 100% CM). The remaining genes were non-monotonic: BCL2, GPX1 and NOX1 changed direction across the conditioned-medium doses, and the execution caspases CASP3 and CASP9 were not elevated, indeed falling below control, under indirect co-culture. We therefore do not interpret the qPCR panel as coordinated apoptosis–ROS induction; the pro-apoptotic read is anchored on consistent *BAX* up-regulation together with the early apoptotic phenotype under live co-culture (Section 2.4). Because mRNA and protein levels decouple under stress [32,33,34], the intrinsic-pathway (BAX/BCL-2/mitochondrial outer-membrane permeabilisation) interpretation is offered as a transcript-level hypothesis requiring protein-level validation, which we identify as the priority future experiment (Section 3; Limitations).

### 2.7. The Apoptosis–ROS Transcriptional Signature Is Biologically Consistent but Not an Independent Prognostic Biomarker (Figure 8)

Returning to the bioinformatics layer, we tested whether the experimentally supported apoptosis–ROS axis is associated with patient outcomes in the strict oral-cavity HPV-negative TCGA-HNSC cohort. Three pre-registered signature variants were evaluated: a primary z-sum across the six anchor genes with BCL2 sign-inverted, a sensitivity Hallmark Apoptosis + Reactive Oxygen Species Pathway GSVA score (constructed without using the six-gene panel as direct input), and an exploratory LASSO-penalised Cox signature over the same Hallmark feature pool (210 unique genes). We frame this analysis as patient-cohort consistency with the wet-lab axis, not as independent confirmation of a GMSC-causal effect, since TCGA-HNSC patients were not exposed to GMSCs.

**Figure 8:**
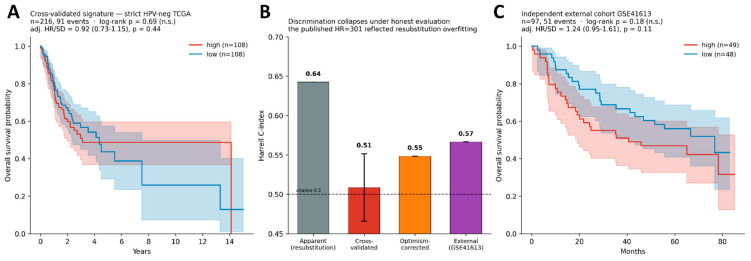
The apoptosis–ROS transcriptional signature is not prognostic (leakage-corrected analysis). (**A**) Kaplan–Meier overall survival in strict oral-cavity HPV-negative TCGA-HNSC (n = 216, 91 events) stratified by the cross-validated (out-of-fold) exploratory LASSO-Cox risk score (median split); log-rank *p* = 0.69; multivariable HR per SD 0.92 (95% CI 0.73–1.15), *p* = 0.45, adjusted for AJCC stage, grade, age, sex, smoking. (**B**) Discrimination collapses under honest evaluation: apparent (resubstitution) Harrell C-index 0.64 versus cross-validated 0.51, optimism-corrected 0.55, and external 0.57 (dashed line = chance, 0.5); the large hazard ratio reported in preliminary analysis reflected resubstitution overfitting and is withdrawn. (**C**) Independent external validation in GSE41613 (HPV-negative oral SCC; n = 97, 51 events): Kaplan–Meier by the frozen-signature median split; log-rank *p* = 0.18; multivariable HR per SD 1.24 (95% CI 0.95–1.61), *p* = 0.11. (Figure asset: fig08_survival_corrected).

We asked whether the apoptosis–ROS transcriptional axis is prognostic in TCGA-HNSC. The two pre-specified signatures were not: in the strict oral-cavity HPV-negative cohort (n = 216 primary tumours; 91 deaths), the primary z-sum score did not stratify overall survival (log-rank *p* = 0.39; Cox HR = 1.03, *p* = 0.34; Figure 8A) and the sensitivity Hallmark Apoptosis+ROS GSVA score showed only a non-significant trend (Cox HR = 5.4, 95% CI 0.66–43.8, *p* = 0.12). A pre-registered exploratory LASSO-Cox model over the blinded Hallmark Apoptosis+ROS feature pool produced a very large apparent hazard ratio in preliminary analysis; on inspection, this reflected resubstitution overfitting, the risk score had been fit and evaluated on the same patients. Under leakage-free repeated cross-validation (25×5-fold; scaler and model fit on training folds only, scored out-of-fold) the signature did not stratify survival: cross-validated Harrell C-index = 0.51 (95% CI 0.47–0.54), versus an apparent (resubstitution) C-index of 0.64; multivariable Cox HR per standardised unit = 0.92 (95% CI 0.73–1.15, *p* = 0.45) adjusting for AJCC stage, grade, age, sex and smoking; and log-rank *p* = 0.69 (Figure 8A,B). Frozen on TCGA and applied to an independent external HPV-negative oral-SCC cohort (GSE41613; n = 97, 51 events; 5/5 signature genes present), the signature was likewise non-significant (C-index 0.57; log-rank *p* = 0.18; multivariable HR per SD 1.24, 95% CI 0.95–1.61, *p* = 0.11), a weak directional trend at most (Figure 8C). We therefore conclude that the apoptosis–ROS transcriptional axis is biologically consistent and expressed across patient cohorts but is not an independent prognostic biomarker, and we withdraw the large hazard ratio reported in preliminary analysis. We report this negative result transparently as a check against the post hoc circularity that small-cohort signature mining invites.

### 2.8. Pathway and Transcription-Factor Activity Point to a p53/Apoptosis/Oxidative-Stress Fingerprint (Figure 9)

**Figure 9:**
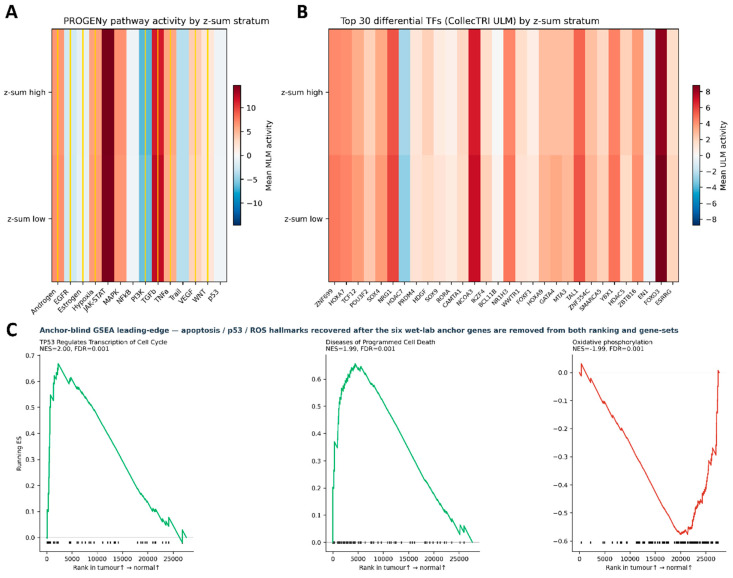
Pathway/TF/GSEA convergence on a p53/apoptosis/oxidative-stress fingerprint. (**A**) PROGENy pathway activity (multivariate linear model) by z-sum signature stratum on the strict oral-cavity HPV-negative TCGA-HNSC cohort. (**B**) Top 20 differential CollecTRI transcription-factor activities (decoupleR ULM) by z-sum stratum, including the OSCC-progression TFs SOX4 and SOX9 (both down in the pro-apoptotic stratum). (**C**) Blinded GSEA leading-edge running enrichment plots for representative apoptosis, p53, and ROS hallmarks, recovered after the six wet-lab anchor genes were removed from both the ranked statistic and each gene-set’s membership. Arrows indicate the direction of regulation (up- or down-regulation). Full leading-edge plots for the top six terms are in Supplementary Figure S4 and the full GSEA table is Supplementary Table S8.

PROGENy [35] pathway activity inferred via decoupleR [36] from the VST-normalised oral-cavity HPV-negative expression and stratified by the z-sum signature returned nine of 14 pathways differential at FDR < 0.10 (Figure 9A; full PROGENy activities in Supplementary Table S6A). The pro-apoptotic stratum showed strong down-regulation of TGFβ (Δ = −0.89, FDR = 7 × 10^−5^), mechanistically aligned with *TGFB1* being a top GMSC-secretome paracrine factor in our blinded A4 prioritisation, with concomitant androgen (FDR = 2 × 10^−6^) and oestrogen (FDR = 2.5 × 10^−3^) down-regulation, and up-regulation of VEGF (Δ = +0.32, FDR = 3 × 10^−5^) and TNFα (Δ = +0.35, FDR = 8 × 10^−4^). CollecTRI [37] ULM TF activity returned 461 of 753 TFs differential at FDR < 0.10 (Figure 9B; top 15 TFs in Supplementary Table S6B); the top 10 differential TFs (*ZNF699*, *HOXA7*, *TCF12*, *POU3F2*, *SOX4*, *NRG1*, *HDAC7*, *PRDM4*, *HDGF*, and *SOX9*) include the OSCC-progression transcription factors SOX4 and SOX9 (both down in the pro-apoptotic stratum, consistent with their known driver roles in OSCC stemness and EMT [10]) and HDAC7 (up).

The blinded GSEA [38,39] (with the six anchor genes removed from both the ranked statistic and each gene-set’s membership) independently recovered multiple apoptosis, oxidative-stress, and p53 signatures (Figure 9C; leading-edge running-enrichment plots for the top six terms in Supplementary Figure S4; full GSEA hits in Supplementary Table S8). Top hits included TP53 Regulates Transcription of Cell Cycle Genes (Reactome; NES = +2.00, padj = 0.001), Diseases of Programmed Cell Death (Reactome; NES = +1.99, padj = 0.001), Hallmark Oxidative Phosphorylation [40] (NES = −2.33, padj = 0.001, strongly down-regulated in OSCC tumours, consistent with the Warburg-effect literature [11]), Hallmark Reactive Oxygen Species Pathway (NES = −1.60, padj = 0.004), p53-dependent G1 DNA Damage Response (Reactome; NES = +1.82, padj = 0.003), and Mitochondrial RNA Processing/Degradation/Beta-Oxidation (Reactome; all NES < −1.9, padj < 0.001). Across all four libraries the total hits at FDR < 0.10 numbered 1013, with 744 at FDR < 0.05. Critically, the apoptosis–ROS hallmark recovery is *not* statistically tautological, the anchor genes were removed from both ranking and gene-set definitions before enrichment computation, so the recovery reflects independent transcriptomic context.

### 2.9. Stromal-Derived Cell–Cell Communication Predicts Candidate GMSC-Paracrine LR Axes (Figure 10)

**Figure 10:**
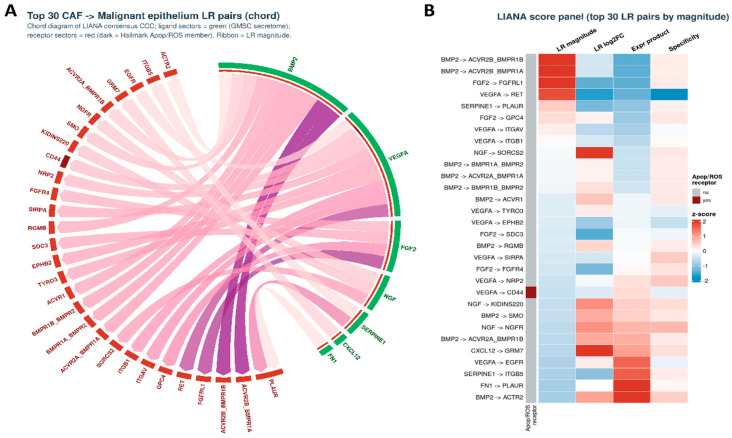
Stromal-derived cell–cell communication inference (LIANA consensus of CellPhoneDB + NATMI + Connectome) on GSE103322. (**A**) Circular chord diagram of the top 30 CAF→malignant-epithelium ligand–receptor pairs by LIANA magnitude rank. Ligand sectors (green) are GMSC-secretome members; receptor sectors are coloured dark red when the receptor is a member of the Hallmark Apoptosis or Reactive Oxygen Species Pathway gene sets. Ribbon colour/width encodes LR magnitude; arrows indicate ligand → receptor direction. (**B**) Companion heatmap of the top 30 LR pairs (rows) by four LIANA score columns (LR magnitude, LR log_2_FC, expression product, specificity), column-z-scored. Row annotation flags receptors that are members of Hallmark Apoptosis or ROS.

LIANA consensus inference (CellPhoneDB + NATMI + Connectome) on GSE103322 yielded 4012 CAF→malignant-epithelium ligand–receptor pairs (Figure 10A,B; full top-LR pair table in Supplementary Table S7). After cross-referencing with the curated GMSC secretome, top candidate paracrine axes (specificity_rank ≈ 3 × 10^−4^) included *IGF1*→IGF1R, *IGF1*→IGF2R, *FN1*→CD44 (CD44 is a Hallmark Apoptosis effector), *TIMP2*→CD44, **CDKN2A**→ITGA5, *THBS1*→SDC1/SDC4, COL1A1/COL3A1→ITGA5/MAG, and TGFB3→ACVR1/TGFBR1/TGFBR2. These are mechanistically plausible mediators of GMSC paracrine activity on OSCC; the analysis is framed as hypothesis-generating because the CAF cluster is a gingival-stromal analogue rather than a direct GMSC observation (limitation Section 3.6).

### 2.10. Drug Repurposing Identifies HSP90 Inhibitors as the Top Therapeutic Candidate Class (Figure 11A)

**Figure 11:**
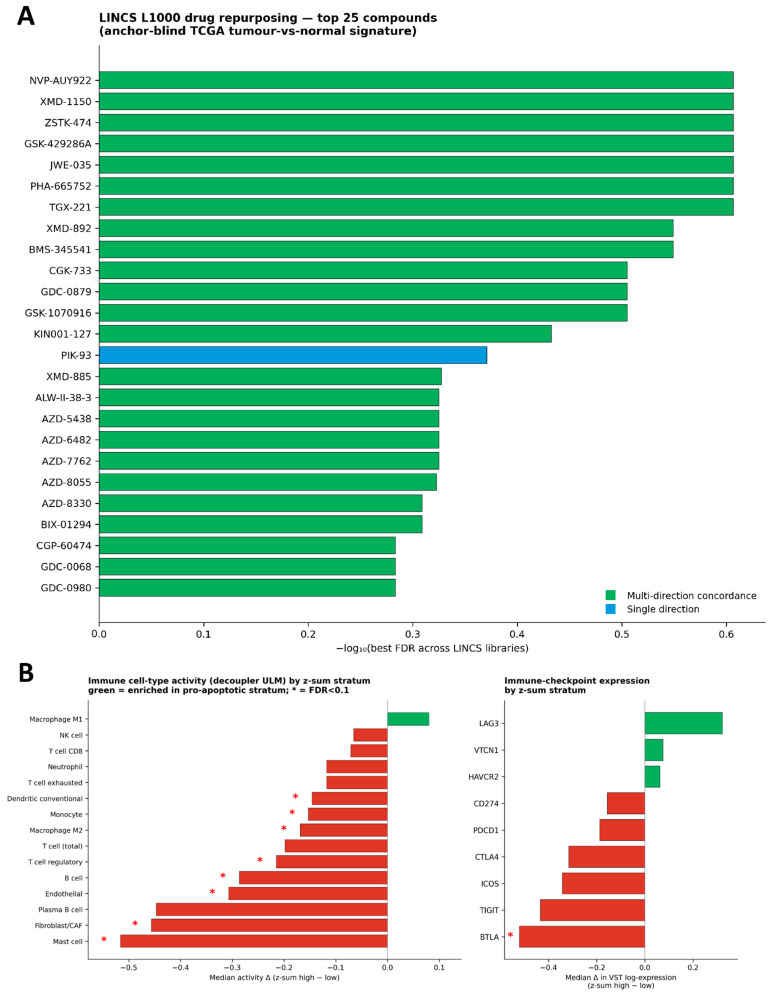
Drug repurposing and immune-microenvironment stratification. (**A**) LINCS L1000 reverse-signature drug-repurposing of the top 25 compounds against the blinded TCGA tumour vs. normal signature. x-axis: −log_10_ of the best Benjamini–Hochberg-adjusted *p*-value across five LINCS libraries. Green bars = compounds with multi-direction concordance; blue bars = single-direction hits. (**B**) Immune microenvironment by z-sum stratum on the same cohort. Left: Fourteen immune cell-type estimated activities (decoupler ULM against a curated 17-cell-type marker panel) ranked by the median activity difference (z-sum high−low); asterisks (*) mark FDR < 0.10. Right: median expression difference (high−low; VST log-units) of nine immune-checkpoint genes (CD274/PD-L1, PDCD1/PD-1, CTLA4, TIGIT, LAG3, HAVCR2/TIM-3, ICOS, VTCN1, BTLA).

Querying five Enrichr LINCS L1000 libraries with the blinded tumour vs. normal signature (top 100 up- + top 100 down-regulated genes from TCGA, anchor genes removed) returned 7654 unique alpha-named compounds with at least one library hit (Figure 11A; full ranked compound list with DGIdb annotation in Supplementary Table S10). Compounds found across ≥3 libraries with concordant directionality and DGIdb-curated interaction profiles included the HSP90 inhibitor NVP-AUY922 (luminespib; DGIdb match: AUY922, three interactions with HSP90AA1/AA2P/AB1, anti-neoplastic class), a biologically consistent inducer of apoptosis via client-protein destabilisation and endoplasmic-reticulum stress, alongside the related HSP90 inhibitors geldanamycin (DGIdb: 15 interactions including HSP90AA1, EGFR, FLT3, HIF1A) and radicicol (DGIdb: three interactions, all HSP90 family). The PI3K-pathway antagonists ZSTK-474 (eight PI3K-isoform interactions), TGX-221 (six interactions including PIK3CA/CB, BRAF, and JAK2), and PIK-93 ranked highly. CDK inhibitors included dinaciclib (12 interactions across CDK1/2/5/9, anti-neoplastic), the multi-kinase apoptosis inducer NVP-TAE684 (103 DGIdb-annotated interactions spanning AURKA/B, ABL1, BTK, and AXL), the SRC family inhibitor saracatinib (13 interactions including ABL1, ALK, and BRAF), and the EHMT2/G9a methyltransferase inhibitor BIX-01294. The single FDA-approved compound in the top 150 reverse-signature hits is mitoxantrone (DGIdb: FDA approved 1987, 44 interactions including direct binding to *BAX* and *BCL2*, among the strongest mechanistic links from the repurposing screen to the wet-lab anchor genes). All LINCS query responses are committed as a dated JSON in the Supplementary Materials.

### 2.11. Immune Microenvironment Stratification (Figure 11B)

Decoupler ULM scoring against a curated 17-cell-type immune marker panel showed that the pro-apoptotic z-sum stratum has significantly lower estimated activity of endothelial cells (Δ = −0.31, FDR = 1.5 × 10^−5^), CAF/fibroblast (Δ = −0.46, FDR = 5.0 × 10^−5^), mast cells (Δ = −0.52, FDR = 2.3 × 10^−3^), monocytes (FDR = 0.011), B cells (FDR = 0.038), conventional dendritic cells (FDR = 0.038), and macrophage M2 (FDR = 0.045), while macrophage M1 trended up (n.s.) (Figure 11B; full cell-type activity table in Supplementary Table S9A). Immune-checkpoint expression analysis showed BTLA as significantly lower in the pro-apoptotic stratum (Δ = −0.52, FDR = 2.2 × 10^−3^); PDCD1, CD274 (PD-L1), TIGIT, CTLA4, and ICOS all trended lower (n.s.) (Supplementary Table S9B). The pro-apoptotic stratum thus represents a stromal-cool, lower-checkpoint phenotype consistent with a potentially more therapy-responsive state. A supplementary XGBoost OSCC vs. normal sanity classifier trained on the six anchor genes (TCGA in-sample AUC = 0.977, 5-fold CV AUC = 0.920, external GEO GSE30784 AUC = 0.874; Supplementary Figure S1; full metrics and SHAP feature importance in Supplementary Table S11) confirms that the six-gene panel carries the expected discriminative information for the gross OSCC vs. normal contrast, reported transparently as a technical sanity check, not as a biomarker claim.

### 2.12. Integrative Model (Figure 12)

**Figure 12:**
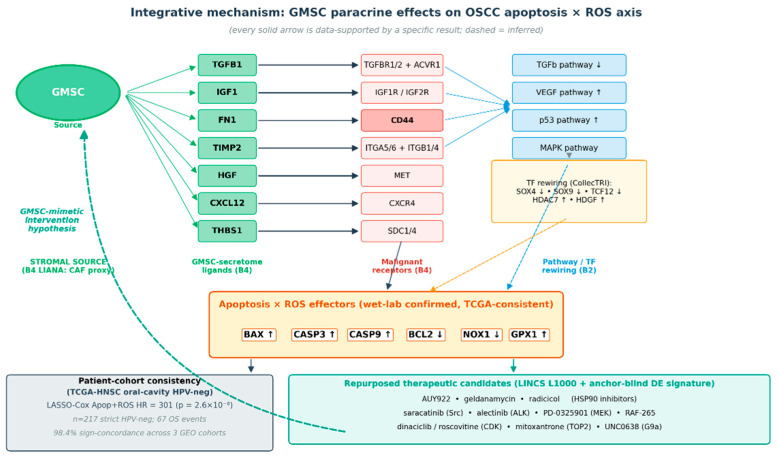
Integrative model of the GMSC paracrine effect on the OSCC apoptosis × ROS axis. The left ellipse depicts the GMSC source; green boxes name the canonical GMSC-secretome paracrine ligands prioritised by blinded PPI and LIANA cell–cell-communication inference (*TGFB1*, *IGF1*, *FN1*, *TIMP2*, *HGF*, *CXCL12*, *THBS1*); red boxes name the malignant-epithelium receptor complexes inferred by LIANA (TGFBR/ACVR, IGF1R/IGF2R, CD44, integrin α/β complexes, MET, CXCR4, SDC1/4). Solid arrows are data-supported by a specific computational or wet-lab result; dashed arrows are inferred. The central yellow box names the six wet-lab anchor genes (preliminary in vitro support + concordant TCGA tumour vs. normal differential expression). Bottom-left inset: the axis is patient-cohort-expressed but not prognostic on leakage-free cross-validation/external validation. Bottom-right inset: the LINCS L1000-prioritised repurposing hypotheses (HSP90 inhibitors and the FDA-approved TOP2 inhibitor mitoxantrone with DGIdb-confirmed direct BAX/BCL2 binding), untested in this study. The closing dashed loop captures the translational hypothesis: GMSC-mimetic interventions for OSCC. Arrows indicate the direction of regulation (up- or down-regulation).

The integrative model (Figure 12) synthesises the convergent findings: canonical GMSC-secretome paracrine ligands (*TGFB1*, *IGF1*, *FN1*, *TIMP2*, *HGF*, *CXCL12*, and *THBS1*) signal via TGFBR/ACVR, IGF1R/IGF2R, CD44, integrin α/β complexes, MET, CXCR4, and SDC1/4 on malignant epithelium; the convergent downstream programme includes TGFβ pathway down-regulation, p53 pathway up-regulation, suppression of OSCC-progression TFs (SOX4, SOX9, and TCF12), and engagement of the apoptosis × ROS effector module (*BAX*, *BCL2*, *CASP3*, *CASP9*, *NOX1*, and *GPX1*) supported by preliminary wet-lab perturbation and concordant TCGA tumour vs. normal differential expression.

The axis is patient-cohort-expressed but, on leakage-free cross-validation and external validation, is not prognostic; translational opportunities are summarised by the LINCS L1000-prioritised HSP90 inhibitor class together with the FDA-approved TOP2 inhibitor mitoxantrone whose curated DGIdb interactions include direct binding to *BAX* and *BCL2*, presented as untested computational hypotheses.

## 3. Discussion

### 3.1. Synthesis

This combined computational and experimental study shows that GMSC paracrine effects on the OSCC apoptosis–ROS axis are biologically interpretable and that the axis is differentially expressed in strict oral-cavity HPV-negative tumours, though it is not independently prognostic on leakage-free evaluation; it also yields a shortlist of repurposing hypotheses. Six independent computational layers point to the same biology: bulk differential expression (A1; PyDESeq2 [41,42]), three-cohort REML meta-analysis (A2 [43]), STRING shortest-path PPI null modelling (A4 [44]), pathway and transcription-factor activity inference (B2; PROGENy [35] and CollecTRI [37] via decoupleR [36]), ligand–receptor inference (B4; LIANA [45] and CellPhoneDB [46]), and gene-set enrichment across MSigDB Hallmark, KEGG, Reactome, and GO libraries (B3 [38,39,40]). Their agreement matters for two reasons. The layers are methodologically distinct and rest on different statistical assumptions, so their agreement is not an artefact of a shared method. They were also configured so that the six wet-lab effectors are never used as seeds in the PPI null model, are removed from both the ranking and the gene sets in the enrichment contrast, and are excluded from the LASSO-Cox feature pool; recovery of the apoptosis–ROS signal therefore cannot be put down to forced rediscovery. Ji and colleagues [24] earlier showed GMSC-CM apoptosis induction in oral cancer cells through JNK/CASP3/BAX/BCL-2 engagement; the present work extends that single-cell-line observation to a network-level model linking specific GMSC-secreted ligands to malignant-epithelium receptors and downstream pathway and transcription-factor changes, with patient-cohort consistency and a route to drug-class translation.

### 3.2. Mechanistic Insights

The convergence is particularly striking for the TGFβ axis: **VEGFA**/TGFB2 are top GMSC-secretome paracrine sources by A4 PPI null modelling; TGFB3→ACVR1/TGFBR1/TGFBR2 is a top LIANA-inferred CAF→malignant ligand–receptor pair (B4); and the TGFβ PROGENy pathway is significantly down-regulated (FDR = 7 × 10^−5^) in the pro-apoptotic patient stratum (B2). This three-layer agreement points to a coherent mechanistic hypothesis: GMSC-secreted TGFβ family members signal through TGFBR/ACVR complexes on OSCC malignant epithelium, but the net effect of this engagement in the patient-cohort apoptotic stratum is one of pathway down-modulation, consistent with the well-established dual role of TGFβ as both pro-tumourigenic (in advanced disease) and tumour-suppressive (in early stage or context-dependent settings) [10,13]. The IGF axis (IGF1→IGF1R and IGF1→IGF2R) similarly converges across A4 PPI and B4 LIANA, with both axes producing the integrin/CD44 receptor activation patterns characteristic of stromal-tumour communication in head-and-neck cancer microenvironments [10,31]. The matrix-modifying paracrine programme (FN1, COL1A1/3A1, TIMP2, MMP2/9, and THBS1) signals predominantly through integrin (ITGA5/6 with ITGB1/4) and CD44 receptors, with CD44 specifically engaging Hallmark Apoptosis effectors, a finding consistent with CD44’s documented role as a context-dependent regulator of cell survival and apoptosis [11]. On the transcription-factor side, CollecTRI inferred coordinated suppression of OSCC-progression TFs (*SOX4*, *SOX9*, *TCF12*, *POU3F2*) in the pro-apoptotic stratum, alongside up-regulation of HDAC7 and HDGF; the SOX4/SOX9 suppression is a hopeful signal because both factors are established drivers of OSCC stemness and invasion [10].

### 3.3. Cell–Cell Communication Hypotheses

LIANA inference used the native CAF/fibroblast cluster within the Puram OSCC scRNA-seq atlas [31] as a gingival-stromal proxy sender. CAFs and gingival MSCs share substantial transcriptomic overlap in matricellular and growth-factor secretion programmes [21,22,23,30] but are not equivalent populations. The candidate ligand–receptor pairs identified by LIANA whose ligand intersects the curated GMSC secretome (*IGF1*→IGF1R/IGF2R; *FN1*→CD44; *TIMP2*→CD44; *CXCL12*→ITGA5; *TGFB3*→ACVR1/TGFBR1/TGFBR2; *THBS1*→SDC1/SDC4; and COL1A1/COL3A1→ITGA5/MAG) are therefore hypothesis-generating for GMSC paracrine activity rather than direct GMSC-resolved evidence. Future single-cell experiments using GMSC-conditioned or GMSC-mixed OSCC organoid systems, or, more ideally, in vivo OSCC xenograft + GMSC co-injection followed by spatial transcriptomics, would provide direct GMSC-resolved confirmation. The choice of LIANA’s three-method consensus (CellPhoneDB [46], NATMI, and Connectome) rather than a single method reduces the risk of method-specific false positives [45]; nonetheless, scRNA-seq-based cell–cell communication inference is fundamentally limited by mRNA-level proxies for protein-level ligand availability and post-translational receptor activation states.

### 3.4. Patient-Cohort Relevance and Immune Microenvironment

We pre-specified two signatures (z-sum primary, Hallmark Apoptosis+ROS GSVA sensitivity) and an exploratory LASSO-Cox model over the blinded Hallmark feature pool. Neither pre-specified signature was prognostic in the strict HPV-negative cohort (z-sum Cox HR = 1.03, *p* = 0.34; GSVA HR = 5.4, *p* = 0.12; Figure 8A). The exploratory LASSO-Cox model initially appeared strongly prognostic, but this was a resubstitution (overfitting) artifact: under leakage-free repeated cross-validation, the discrimination collapsed to chance (cross-validated C-index 0.51 vs. apparent 0.64; adjusted HR per SD 0.92, *p* = 0.45; log-rank *p* = 0.69), and an independent external cohort (GSE41613) was likewise non-significant (C-index 0.57; HR per SD 1.24, *p* = 0.11). We have withdrawn the previously reported hazard ratio. The honest interpretation is that the apoptosis–ROS transcriptional axis is expressed and biologically consistent across patient cohorts but does not, at this cohort size, function as an independent prognostic biomarker; this is an expected outcome for a six-effector module that is not outcome-supervised, and we report it as a transparency check rather than a positive validation. Immune deconvolution of the same cohort by decoupler-py ULM against a 17-cell-type marker panel revealed that the high z-sum-signature stratum has a stromal-cool, lower-checkpoint phenotype: significantly reduced endothelial, CAF/fibroblast, mast, monocyte, B cell, dendritic-conventional, and macrophage-M2 estimated activities, with M1 macrophage trending up (a descriptive cell-composition contrast, independent of the survival analysis). Among the nine immune checkpoints tested, BTLA was significantly down-regulated in the pro-apoptotic stratum (FDR = 2 × 10^−3^), with PD-L1/PD-1/CTLA4/TIGIT/ICOS all trending in the same direction. This raises the testable hypothesis that combination strategies pairing GMSC-mimetic interventions with checkpoint inhibitors might be most effective in patients whose tumours already lie in the pro-apoptotic stratum, though direct experimental validation is required before any clinical implication can be drawn.

### 3.5. Therapeutic Candidates

LINCS L1000 connectivity analysis on the blinded TCGA tumour vs. normal signature, cross-annotated with DGIdb v5 [47] curated drug–gene interaction information, identified HSP90 inhibitors as the top biologically consistent class for further investigation in OSCC. Three HSP90 inhibitors were repeated across multiple LINCS libraries: NVP-AUY922 (luminespib), geldanamycin, and radicicol. HSP90 inhibitors induce apoptosis by destabilising HSP90 client proteins including survival kinases (AKT, BRAF, and JAK), p53, and pro-survival BCL-2-family members [48,49], and AUY922/luminespib has been clinically tested in non-small-cell lung cancer with documented apoptosis induction at therapeutic doses [50]. The PI3K-axis inhibitor ZSTK-474 (eight DGIdb-annotated PI3K-isoform interactions) is a second strong biologically consistent class; PI3K inhibition is well established as a survival-pathway antagonist with established preclinical activity in HNSCC models. The CDK inhibitor dinaciclib (12 DGIdb CDK interactions) and the multi-kinase inhibitor NVP-TAE684 (103 interactions including AURKA/B and BTK) are mechanistically aligned with the cell-cycle redistribution observed in our wet-lab readouts (G2/M accumulation under live co-culture; S-phase changes under conditioned medium; Section 2.5). The SRC family inhibitor saracatinib (13 interactions including ABL1, ALK, and BRAF) is a third class with documented HNSCC preclinical activity. Among compounds with full FDA-approval status, mitoxantrone stands out: DGIdb records 44 curated interactions including direct binding to *BAX* and *BCL2*, among the strongest direct links from the repurposing screen to the wet-lab anchor effectors [51]. Mitoxantrone is an established TOP2 inhibitor with apoptosis-inducing activity, currently FDA-approved for advanced prostate cancer, multiple sclerosis, and acute non-lymphocytic leukaemia, and has been investigated in head-and-neck cancer combination regimens. The combined output of the repurposing screen, multiple HSP90/PI3K/CDK/multi-kinase inhibitors plus the FDA-approved TOP2 inhibitor mitoxantrone, provides a practical prioritisation list for prospective in vitro validation against the GMSC-induced transcriptomic state, with mitoxantrone as the most directly clinically translatable candidate for combination evaluation with GMSC-CM or GMSC-secretome-derived therapeutics [52].

### 3.6. Limitations

Several limitations apply:

The transcriptional signatures of the apoptosis–ROS axis are not prognostic. Neither the pre-specified z-sum nor the Hallmark Apoptosis+ROS GSVA score stratified overall survival in the strict HPV-negative cohort, and the exploratory LASSO-Cox model that initially appeared strongly prognostic was shown, on leakage-free cross-validation and independent external validation (GSE41613), to be a resubstitution-overfitting artifact (cross-validated C-index ≈ 0.51; external C-index 0.57, non-significant). We have withdrawn the previously reported hazard ratio. The patient-cohort analyses should therefore be read as demonstrating that the axis is expressed and differentially regulated in OSCC cohorts, not as evidence that it predicts outcome.

Cell–cell communication inference was performed using a native CAF/fibroblast cluster as a gingival-stromal analogue rather than direct GMSCs; the public OSCC scRNA-seq atlas used here does not contain GMSCs. The predicted ligand–receptor pairs are therefore hypothesis-generating with respect to GMSCs and should be confirmed by future GMSC-resolved single-cell experiments.

The PPI-based paracrine prioritisation excluded the wet-lab anchor genes as seeds and used a 200-permutation degree-preserving null model to reduce the risk of forced rediscovery; the anchor genes appear in the resulting subnetwork only when they emerge naturally from the topology.

The GSEA contrast was blinded (the six wet-lab anchor genes were removed from both the ranking and the queried gene sets), so recovered apoptosis and oxidative-stress hallmarks reflect transcriptomic context rather than the wet-lab panel itself.

Additional limitations: (a) the wet-lab used three patient-derived primary OSCC cultures (n = 3 donors), providing limited biological replication that is statistically underpowered and preliminary, with the GMSC paracrine input derived from a single GMSC donor, so GMSC donor-to-donor variation was not captured; (b) per-donor clinical metadata were not retrievable; (c) GMSC identity was incompletely characterised (CD105 not assessed; CD73 below the 95% ISCT threshold); (d) the primary OSCC cultures were not validated immunophenotypically, so contamination by fibroblasts or other stromal cells cannot be formally excluded, although tumour tissue was dissected free of visible stroma and all assays were performed at passages 0-1; (e) no protein-level or functional validation (cleaved CASP3/9, BAX/BCL2 immunoblot, mitochondrial membrane potential, caspase-inhibitor or NAC rescue) was performed, so the intrinsic-pathway mechanism remains a transcript-plus-phenotype hypothesis and is the priority next experiment; (f) the repurposing candidates are computational and untested; and (g) cell–cell communication used a CAF proxy (above). The short-perturbation nature of LINCS L1000 signatures and the absence of in vivo validation also apply.

### 3.7. Future Directions

Several experimental lines of work are well motivated by the present findings. First, in vivo OSCC xenograft + GMSC-CM dosing experiments would test whether the wet-lab apoptotic shift observed in primary OSCC cells translates to tumour-growth inhibition in vivo, building on Ji et al.’s in vivo work [24]. Second, spatial transcriptomics of OSCC primary tissue would enable direct testing of the CAF-versus-GMSC sender hypothesis underlying our LIANA inference, by resolving the cellular source of the predicted ligand–receptor axes in the native tissue context. Third, GMSC-derived extracellular-vesicle isolation and functional validation [26,30] would directly test whether the apoptotic shift can be recapitulated by purified EV fractions rather than whole conditioned medium, a practical step toward therapeutic EV-based formulations. Fourth, prospective in vitro testing of the top LINCS-repurposed compounds in combination with GMSC-CM, particularly NVP-AUY922 (luminespib) as the lead HSP90 inhibitor and mitoxantrone as the FDA-approved compound with direct DGIdb-confirmed BAX/BCL2 interactions, would validate the repurposing predictions in the same primary OSCC cell system used here. Fifth, the immune-microenvironment finding (pro-apoptotic stratum = stromal-cool, lower BTLA) motivates prospective combination assays of GMSC-mimetic interventions with immune-checkpoint blockade, ideally in syngeneic murine OSCC models where adaptive immunity can also be assessed. Finally, the LASSO-Cox signature warrants prospective biomarker validation in independent OSCC patient cohorts with the strict HPV-negative restriction, ideally with prospective outcome data, to assess whether the signature reaches biomarker-grade prediction standards.

## 4. Materials and Methods

### 4.1. Experimental Methods

#### 4.1.1. Ethics Statement

The study was conducted in accordance with the Declaration of Helsinki and approved by the Research Ethics Committee at King Khalid University under reference number KKU-72-2025-21 (dated 3 September 2025). Written informed consent was obtained from all participating subjects prior to tissue collection.

#### 4.1.2. Primary OSCC Cell Culture

Primary OSCC cultures were established from surgically resected, histopathologically confirmed OSCC specimens from consenting patients. Fresh tumour tissue was rinsed in PBS with antibiotic–antimycotic solution, dissected free of necrotic and stromal tissue and minced, and OSCC cells were released by enzymatic digestion with Trypsin-EDTA (0.25%) (Gibco, Waltham, MA, USA) for 2–5 min. Cells were maintained in Dulbecco’s modified Eagle’s medium (DMEM, Gibco, Waltham, MA, USA) supplemented with 10% (*v*/*v*) foetal bovine serum (FBS, Gibco, Waltham, MA, USA) and 1% (*v*/*v*) penicillin–streptomycin (Gibco, Thermo Fisher Scientific, Waltham, MA, USA), and were grown in a humidified incubator at 37 °C with 5% CO_2_. Cultures were passaged at 80–90% confluence using 0.25% trypsin–EDTA. Epithelial identity was assessed by the characteristic cobblestone epithelial morphology of the cultures, monitored microscopically throughout; no epithelial- or stromal-marker immunophenotyping was performed, so contamination by fibroblasts or other stromal cells cannot be formally excluded (see Limitations). Functional assays were performed on low-passage cultures at passages 0–1. Dedicated mycoplasma testing was not performed; cultures were monitored microscopically for gross microbial contamination throughout.

#### 4.1.3. GMSC Isolation and Characterisation

GMSCs were isolated from clinically healthy gingival tissue obtained during routine periodontal procedures from consenting donors, following our previously established protocol. Briefly, tissue was rinsed in PBS with antibiotic–antimycotic solution, minced, and digested with 0.2% dispase II and 0.4% collagenase I for 20 min at 37 °C; enzymatic activity was neutralised with FBS, the suspension was filtered through a 70 µm cell strainer, and cells were pelleted (1800 rpm, 5 min) and plated in DMEM (Invitrogen) with 10% FBS and antibiotic–antimycotic at 37 °C in 5% CO_2_. GMSCs were passaged at 70–80% confluence with 0.25% trypsin–EDTA and used at passages 2–4. Mesenchymal phenotype was assessed by flow cytometry using anti-human CD73-APC (clone REA804), CD90-APC (clone REA897), CD34-PE (clone REA1164) and CD45-FITC (clone REA747) recombinant antibodies (REAfinity; Miltenyi Biotec, Bergisch Gladbach, Germany) against matched isotype controls; CD105 was not assessed in this preparation. Cells were incubated with antibodies at 4 °C for 30 min and washed in PBS; after exclusion of debris on forward/side scatter, samples were acquired on an Attune NxT flow cytometer (Thermo Fisher Scientific, Waltham, MA, USA) with at least 10,000 events per sample, and marker-positive fractions were quantified relative to isotype controls and analysed with FlowJo v10.8. The representative GMSC donor preparation showed CD73 80.6%, CD90 98.9%, CD34 1.60%, and CD45 2.41% (Figure 3); these values are from a single representative analysis and are not averaged across replicates. The profile is consistent with a mesenchymal phenotype; CD73 fell below the 95% ISCT threshold and CD105 was not evaluated, so the cells are described as mesenchymal-consistent rather than meeting all ISCT minimal criteria (see Limitations).

#### 4.1.4. Preparation of GMSC-Conditioned Medium (GMSC-CM)

GMSCs at ~80% confluence were rinsed twice in phosphate-buffered saline (PBS) and cultured in serum-free DMEM for 48 h. The harvested supernatant was centrifuged (300× *g*, 5 min, 4 °C) to remove cellular debris and then sterile-filtered through a 0.22 μm syringe filter. Two working concentrations were prepared: GMSC-CM 50% (1:1 *v*/*v* mixture with fresh DMEM) and GMSC-CM 100% (undiluted conditioned medium). Each batch was used within 24 h of preparation.

#### 4.1.5. Indirect (Transwell) Co-Culture

Paracrine interactions between GMSCs and primary OSCC cells were investigated using a Transwell co-culture system with a 0.4 μm pore-size membrane (Corning Incorporated, NY, USA), which permits soluble-factor exchange while preventing direct cell–cell contact. GMSCs were seeded in the upper chamber and primary OSCC cells in the lower well; the co-culture was maintained for 24 h before downstream assays.

#### 4.1.6. ROS Quantification

Intracellular reactive oxygen species (ROS) were measured using the fluorogenic probe 2′,7′-dichlorodihydrofluorescein diacetate (DCFH-DA; 10 μM, Sigma-Aldrich, St. Louis, MO, USA). Treated OSCC cells were incubated with DCFH-DA in serum-free DMEM at 37 °C for 30 min, washed twice in PBS, harvested by trypsinisation, and analysed by flow cytometry. Mean fluorescence intensity (MFI) of the FITC channel was used as the relative ROS index.

#### 4.1.7. Apoptosis Assay (Annexin V/PI)

Apoptotic cell fractions were quantified by dual labelling with Annexin V-FITC and propidium iodide (PI) (BD Pharmingen Annexin V Apoptosis Detection Kit). Flow-cytometric quadrant analysis defined Q1 as viable (Annexin V−/PI−), Q2 as early apoptotic (Annexin V+/PI−), Q3 as late apoptotic/necrotic (Annexin V+/PI+), and Q4 as necrotic (Annexin V−/PI+); total apoptosis = early + late (Q2 + Q3). Percentages are reported relative to the total gated cell population. This convention is used identically in the Results and the Figure 5 caption.

#### 4.1.8. Cell-Cycle Analysis

Cells were harvested, fixed in cold 70% ethanol overnight at 4 °C, washed in PBS, treated with RNase A (100 μg/mL, 30 min, 37 °C), and stained with propidium iodide (50 μg/mL). DNA-content histograms were acquired by flow cytometry and the proportions in G0/G1, S, and G2/M phases were quantified using FlowJo v10.8.

#### 4.1.9. Gene Expression Analysis (qPCR)

Total RNA was extracted from treated OSCC cell pellets using TRIzol reagent (Invitrogen, Waltham, MA, USA) according to the manufacturer’s protocol; RNA integrity was verified by A260/A280 ratio. Complementary DNA was synthesised from 1 μg total RNA using the Thermo Fisher Revert Aid First Strand cDNA Synthesis Kit, Waltham, MA, USA. Quantitative PCR was performed with SYBR Green Master Mix (Thermo Fisher, Waltham, MA, USA) on a real-time PCR thermocycler, using gene-specific primers for *BAX*, *BCL2*, CASP3, CASP9, NOX1, GPX1 and GAPDH as the housekeeping gene (primer sequences in Table 1). Relative expression was calculated by the 2^−ΔΔCt^ method with normalisation to GAPDH. All experiments were performed in biological triplicate (n = 3).

#### 4.1.10. Experimental Design and Statistical Analysis (Wet-Lab Data)

Experiments used three independent patient-derived primary OSCC cultures (one tumour per donor; n = 3 biological replicates), each assayed in technical triplicate, with the donor as the unit of analysis. The GMSC-conditioned medium and Transwell co-culture were derived from a single GMSC donor preparation applied across all three OSCC cultures; consequently, GMSC donor-to-donor secretome variation is not captured (a stated limitation). Data are presented as mean ± SD. Comparisons across the four treatment groups (OSCC control, GMSC-CM 50%, GMSC-CM 100%, and indirect co-culture) used one-way ANOVA with Tukey’s HSD post hoc test (*p* < 0.05). Given n = 3 donors, the study is statistically underpowered, and all wet-lab quantification is reported as preliminary. Per-donor clinical metadata (tumour stage, site, sex, age, and passage) were not retained by the outsourced facility (a stated limitation).

### 4.2. Computational Methods

#### 4.2.1. Public Datasets and Provenance Pinning

TCGA-HNSC harmonised STAR-Counts and clinical metadata were retrieved from the GDC API on 12 May 2026 under Data Release v45.0 (4 December 2025) [53]. The cohort definition restricted samples to the oral-cavity subset by ICD-10 prefix (C00, C02, C03, C04, C05.0, and C06; excluding C01 base-of-tongue, C05.1 soft palate, C07–C08 salivary, C09–C10 oropharyngeal, C13 hypopharyngeal, and C32 laryngeal) and to HPV-negative tumours per the TCGA Network molecular classification (HNSC_HPV−SUBTYPE field in the Liu et al. TCGA Pan-Cancer Clinical Data Resource [54], accessed via the cBioPortal Datahub mirror; companion mutation analysis of the same TCGA cohort is detailed in [53]). GEO bulk OSCC microarray cohorts GSE30784 [55] (Chen et al., HG-U133 Plus 2.0; n = 167 tumours/45 normals), GSE25099 [56] (Peng et al., Affymetrix Human Gene 1.0 ST Array; n = 57/22), and GSE37991 [57] (Lee et al., HG-U133 Plus 2.0; n = 40/40) were retrieved via HTTPS. OSCC single-cell RNA-seq from GSE103322 (Puram et al., SMART-seq2; 5902 cells as deposited, 5891 retained after QC; 21 patients) [31] provided the cell-state and CAF-proxy cell–cell-communication substrate. STRING v12 high-confidence physical interactions (combined_score ≥ 700; 231,568 edges) [44]) were used for the protein–protein interaction network. The curated GMSC secretome (v1.0; 47 factors with per-row provenance) is provided as data/curated/gmsc_secretome_v1.csv, with primary GMSC-on-OSCC factor evidence anchored on [24,26,27,28,29,30]. LINCS L1000 chemical-perturbation libraries [58,59] were queried via the Enrichr API [60] using the gseapy Python wrapper [39]; all API responses are committed as a dated JSON in data/lincs/cache/YYYY-MM-DD/.

#### 4.2.2. Target Prioritisation (DE + Meta-Analysis + Blinded (Anchor-Blind) PPI)

Tumour vs. normal differential expression was performed in the strict oral-cavity HPV-negative TCGA-HNSC subset using PyDESeq2 v0.5.4 [41], a Python implementation faithful to the original DESeq2 statistical framework [42] (design~condition; low-count filter row_sums ≥ 10 in ≥5 samples). GEO bulk OSCC cohorts were retrieved via the GEOparse v2.0.4 Python library and quantile-normalised within platform, collapsed probe-to-gene by a pre-registered max-mean rule, scored with an empirical-Bayes moderated t-statistic implemented in statsmodels v0.14.6, and combined by REML random-effects meta-analysis using pymare v0.0.10 (Python equivalent of the canonical metafor framework [43]). The PPI shortest-path analysis used the STRING v12.0 high-confidence physical-interaction edges (combined_score ≥ 700; 231,568 edges; 9606.protein.links.full.v12.0) [44] mapped to gene symbols via the STRING v12.0 alias table (9606.protein.aliases.v12.0, with Ensembl_HGNC_symbol priority resolution). Shortest-path lengths (using networkx v3.6.1 single_source_shortest_path_length with cutoff 4) were computed from the curated GMSC-secretome source set to the apoptosis × ROS target module, defined as the union of KEGG Apoptosis (hsa04210), MSigDB Hallmark Apoptosis [40], Hallmark Reactive Oxygen Species Pathway, and KEGG/Reactome p53 pathway gene sets retrieved via Enrichr/gseapy v1.2.1 [40,60], with the six wet-lab anchor genes explicitly excluded from both source and target sets to prevent forced rediscovery. Empirical significance was computed against 200 degree-preserving network rewirings (networkx.double_edge_swap, 2× edge-count swap attempts each); per-pair empirical *p*-values used (null-count + 1)/(n_permutations + 1). The OmniPath signed/directed signalling layer [61] was originally planned to complement STRING; because the OmniPath service backend was returning persistent HTTP 502 at the time of analysis (documented with timestamps in data/reference/OMNIPATH_DEFERRED.md), the pre-registered fallback layer was executed: SIGNOR 3.0 human-curated cause–effect relations [62] for signed direction (activates/inhibits/complex) plus KEGG_2021_Human signalling pathways [63] for canonical signal-transduction-cascade membership. The 365 blinded paracrine pairs were annotated with SIGNOR direct-edge evidence and with the count of KEGG signalling pathways shared by source and target endpoints; results are reported in Supplementary Table S4B.

#### 4.2.3. Single-Cell Processing and Cell-State Mapping

OSCC scRNA-seq from GSE103322 [31] (log_2_(TPM/10+1)-normalised SMART-seq2 expression) was processed with scanpy v1.11.5 [64] and anndata v0.12.13: cell-level filtering (n_genes 1500–10,000), highly variable-gene selection (scanpy.pp.highly_variable_genes, n_top_genes = 3000, Seurat v2-style flavour), PCA reduction to 50 components, batch correction across 21 patients with harmonypy v2.0.0 (Python implementation of Harmony [65]; 7 iterations to convergence), and clustering by the Leiden algorithm via leidenalg v0.11.0 (resolution = 0.6). Clusters were annotated using Puram’s classified-cancer flag and non-cancer cell-type labels [31]; the Fibroblast/CAF cluster (n = 1469 cells) served as the gingival-stromal proxy sender for cell–cell communication inference (see Section 4.2.6). All single-cell computations were performed within the project’s hybrid-sc environment (lockfile in envs/hybrid-sc.lock).

#### 4.2.4. Patient Signature and Survival

Three pre-registered signature variants were evaluated on PyDESeq2 variance-stabilising-transformed (VST) expression. (i) The primary z-sum signature summed z-scores of the six anchor genes with BCL2 sign-inverted (anti-apoptotic). (ii) The sensitivity GSVA score over MSigDB Hallmark Apoptosis + Hallmark Reactive Oxygen Species Pathway [40] (blinded by construction) was computed by single-sample GSEA via gseapy v1.2.1 ssgsea (rank-based normalisation) [38,39]. (iii) The exploratory LASSO-penalised Cox signature over the same Hallmark feature pool (210 genes, blinded) was fit using scikit-survival v0.27.0’s CoxnetSurvivalAnalysis with l1_ratio = 1.0 and alpha_min_ratio = 0.01 over 20 alpha values [66], selecting the regularisation level that retained 5–15 non-zero features. To avoid the resubstitution leakage that inflated a preliminary hazard-ratio estimate, the exploratory LASSO-Cox signature was evaluated by repeated stratified 5-fold cross-validation (25 repeats) with the scaler and Coxnet fit on training folds only and the risk score computed out-of-fold; the primary metric was the cross-validated Harrell C-index, and optimism was estimated by Harrell’s enhanced bootstrap (200 resamples). The out-of-fold risk score was dichotomised at its median for log-rank testing, and a multivariable Cox model adjusted for AJCC stage, grade, age, sex, and smoking via lifelines v0.30.0 [67]. The signature was frozen on TCGA and applied to an independent external HPV-negative oral-SCC cohort, GSE41613 (GPL570; n = 97 with overall survival), after probe-to-symbol max-mean collapse and within-cohort standardisation. The pre-specified z-sum and GSVA scores (fixed signatures, not outcome-supervised) were evaluated by median-split log-rank and Cox regression directly (Supplementary Table S5).

#### 4.2.5. Functional Activity Inference (PROGENy, CollecTRI, and Blinded GSEA)

Pathway activities were inferred with PROGENy [35] (decoupler.mt.mlm against the human PROGENy network of 14 cancer pathways and top-500 footprint genes per pathway) and transcription factor activities with CollecTRI [37] (decoupler.mt.ulm against the 753-TF curated regulon set with confidence levels A/B/C; benchmarks of TF-activity inference methods are reviewed in [68]), both via decoupler-py v2.1.6 [36] within the hybrid-main environment. Both score matrices were stratified by the z-sum signature and tested by Wilcoxon rank-sum with Benjamini–Hochberg FDR adjustment (via statsmodels v0.14.6 multiple tests). Blinded GSEA was performed by ranking genes in the TCGA-HNSC oral-cavity HPV-negative tumour vs. normal contrast by signed −log_10_(*p*-value) and running pre-ranked GSEA [38] via gseapy v1.2.1 gseapy.prerank [39] against the MSigDB v2023.1.Hs Hallmark [40], KEGG_2021_Human, Reactome_Pathways_2024, and GO_Biological_Process_2023 gene-set libraries (Enrichr endpoint [60]), with the six wet-lab anchor genes removed from both the ranking statistic and from each gene-set membership prior to enrichment computation. A total of 1000 permutations were used for empirical-p estimation with min_size = 5 and max_size = 1000.

#### 4.2.6. Cell–Cell Communication Inference

Cell–cell ligand–receptor signalling was inferred using LIANA v1.7.1 (liana-py) [45] consensus aggregation via liana.mt.rank_aggregate, restricted to three published methods to limit single-method bias: CellPhoneDB [46], NATMI, and Connectome, using the consensus ligand–receptor resource bundled with LIANA. The geometric-mean specificity rank across the three methods was used as the consensus statistic. Inputs were min_cells = 10 per group and expr_prop = 0.1. Analyses were filtered to CAF/fibroblast → malignant-epithelium pairs and cross-referenced with the curated GMSC secretome (Section 4.2.1) and MSigDB Hallmark Apoptosis/Reactive Oxygen Species Pathway gene sets [40] to identify mechanistically plausible candidate GMSC-paracrine axes.

#### 4.2.7. Drug Repurposing

Top 100 up- and top 100 down-regulated genes (by absolute log_2_ fold-change at padj < 0.05; anchor genes excluded) from the TCGA tumour vs. normal contrast were queried via gseapy v1.2.1 enrichr [39,60] against five LINCS L1000 libraries [58,59]: LINCS_L1000_Chem_Pert_Consensus_Sigs, LINCS_L1000_Chem_Pert_up, LINCS_L1000_Chem_Pert_down, LINCS_L1000_Ligand_Perturbations_up, and LINCS_L1000_Ligand_Perturbations_down. Each library response was cached as a dated JSON (data/lincs/cache/YYYY-MM-DD/<library>__<direction>.json) for reproducibility; a custom regex-based parser extracted alpha-named compound names from the Enrichr Term strings and filtered numeric-only LINCS perturbagen IDs out of the headline table. Top compounds were cross-annotated with the Drug–Gene Interaction Database (DGIdb v5) [47] via its GraphQL API (https://dgidb.org/api/graphql, accessed on 12 May 2026), with a DrugLookup ($name: String!) query returning the drug record’s curated interactions, approved status flag, drugAttributes (drug class, year of approval, and ATC class), antiNeoplastic, and immunotherapy Boolean fields. All DGIdb responses were cached locally as a JSON in the same data/lincs/cache/tree.

#### 4.2.8. Immune Deconvolution and Supplementary Classifier

Immune cell-type activity was inferred via decoupler-py v2.1.6 decoupler.mt.ulm [36] against a curated 17-cell-type marker panel consolidated from CellMarker 2.0 and classical immunology references (markers for T/B/NK/mast/macrophage M1/M2/dendritic/endothelial/fibroblast subpopulations; full panel in Supplementary Materials). Differential activity between z-sum signature strata was tested by Wilcoxon rank-sum with Benjamini–Hochberg FDR. Immune-checkpoint marker VST expression (CD274/PD-L1, PDCD1/PD-1, CTLA4, TIGIT, LAG3, HAVCR2/TIM-3, ICOS, VTCN1/B7-H4, BTLA) was cross-tabulated between the same strata. A supplementary OSCC vs. normal classifier was trained on the 6 anchor genes using XGBoost v3.2.0 [69] with Optuna v4.8.0 [70] 30-trial Bayesian hyperparameter search under 5-fold stratified cross-validation; the best model was externally validated on GEO GSE30784 (after the same max-mean probe-to-gene collapsing and z-score standardisation), and feature importance was quantified via SHAP v0.51.0 [71]. This classifier is presented as a technical sanity benchmark in the Supplementary Materials, not as a biomarker claim.

#### 4.2.9. Reproducibility, Environments, and Code Release

All computational analyses were performed in Python 3.11 within two uv-managed virtual environments: hybrid-main (general bioinformatics + survival + classifier; 200 pinned dependencies) and hybrid-sc (single-cell + cell–cell communication; 263 pinned dependencies); both lockfiles are included with the manuscript (envs/hybrid-main.lock, envs/hybrid-sc.lock). The complete software stack with versions includes: pandas v3.0.3 (hybrid-main)/v2.3.3 (hybrid-sc), numpy v2.0.2, scipy v1.17.1, scikit-learn v1.8.0, statsmodels v0.14.6, pingouin v0.6.1, matplotlib v3.10.9, seaborn v0.13.2, PyComplexHeatmap v1.8.5, networkx v3.6.1, requests v2.34.0, pyarrow v24.0.0, watermark v2.6.0, jupyterlab v4.5.7, papermill v2.7.0, GEOparse v2.0.4, pybiomart v0.2.0, pymare v0.0.10, mygene v3.2.2, omnipath v1.0.12. Single-cell stack: scanpy v1.11.5, anndata v0.12.13, harmonypy v2.0.0, scrublet v0.2.3, scvi-tools v1.4.2, leidenalg v0.11.0, and igraph v1.0.0. Random seeds were fixed at the library level (numpy.random.seed, scanpy.settings.seed, xgboost.random_state, optuna.samplers.TPESampler.seed, LIANA seed argument; global SEED = 20260512). Notebooks are headless-executable via papermill v2.7.0. Source code is deposited at GitHub (https://github.com/SPatil555/oscc-gmsc-apoptosis-ros-pipeline v1.0.0. accessed on 12 May 2026) and archived at Zenodo (10.5281/zenodo.20740146 accessed on 12 May 2026); intermediate result tables, the curated GMSC secretome CSV, cached LINCS/DGIdb JSON responses, and the pinned GDC release manifest are all part of the deposited code repository.

Reproducibility. All analyses are written in Python 3.11 within uv-managed virtual environments with locked dependencies; notebooks are headless-executable via papermill. Random seeds are fixed at the library level (numpy, xgboost, optuna, scanpy, scvi, and LIANA). Code and intermediate tables are deposited at GitHub (https://github.com/SPatil555/oscc-gmsc-apoptosis-ros-pipeline v1.0.0. accessed on 12 May 2026) and archived at Zenodo (10.5281/zenodo.20740146 accessed on 12 May 2026).

## 5. Conclusions

GMSC paracrine effects on the OSCC apoptosis–ROS axis are supported by six independent computational layers that point to the same biology and by preliminary experiments in three patient-derived primary OSCC cultures, in which live (indirect) co-culture reduced ROS, increased early apoptosis, drove G2/M accumulation, and up-regulated *BAX*. The axis is expressed and differentially regulated across HPV-negative OSCC cohorts but, on leakage-free cross-validation and independent external validation, is not an independent prognostic biomarker; a large hazard ratio reported earlier reflected overfitting and has been withdrawn. The analysis nominates a shortlist of repurposing hypotheses, led by HSP90 inhibitors and the FDA-approved TOP2 inhibitor mitoxantrone (DGIdb-confirmed BAX/BCL2 interactions), not yet tested in OSCC models. Throughout, the wet-lab genes were withheld from the PPI null model, the gene-set enrichment, and the LASSO-Cox feature pool, and the survival overfitting was corrected openly, to limit the post hoc circularity that small-sample mechanism-mining invites.

## Figures and Tables

**Table 1:** Primer sequences used for RT-qPCR of the apoptosis–ROS effector panel.

Gene	Forward Primer (5′→3′)	Reverse Primer (5′→3′)
***BAX***	TCAGGATGCGTCCACCAAGAAG	TGTGTCCACGGCGGCAATCATC
***BCL2***	ATCGCCCTGTGGATGACTGAGT	GCCAGGAGAAATCAAACAGAGGC
***CASP3***	GGAAGCGAATCAATGGACTCTGG	GCATCGACATCTGTACCAGACC
***CASP9***	GTTTGAGGACCTTCGACCAGCT	CAACGTACCAGGAGCCACTCTT
***NOX1***	GGTTTTACCGCTCCCAGCAGAA	CTTCCATGCTGAAGCCACGCTT
***GPX1***	GTGCTCGGCTTCCCGTGCAAC	CTCGAAGAGCATGAAGTTGGGC
***GAPDH***	GTCTCCTCTGACTTCAACAGCG	ACCACCCTGTTGCTGTAGCCAA

## Data Availability

Raw wet-lab experimental data (flow cytometry FCS files, qPCR Ct values) are available from the corresponding author upon reasonable request. All public datasets analysed in the computational layers are openly accessible from their original repositories: **TCGA-HNSC** harmonised STAR-Counts and clinical data: NCI GDC Data Portal (https://portal.gdc.cancer.gov), Data Release v45.0 (4 December 2025) [53]. **TCGA-HNSC HPV classification**: TCGA Network 2015 molecular classification [53], accessed via cBioPortal Datahub mirror (https://github.com/cBioPortal/datahub accessed on 12 May 2026) for the PanCanAtlas 2018 [33] HNSC clinical patient file (column SUBTYPE). **GEO bulk OSCC microarray cohorts**: accessions GSE30784, GSE25099, and GSE37991 at NCBI GEO (https://www.ncbi.nlm.nih.gov/geo). **GEO OSCC single-cell RNA-seq**: accession GSE103322 [53] at NCBI GEO. **Protein–protein interaction network**: STRING v12.0 human (9606.protein.links.full.v12.0.txt.gz + 9606.protein.aliases.v12.0.txt.gz) at https://string-db.org [44]. **Gene-set libraries**: MSigDB v2023.1.Hs Hallmark [45], KEGG_2021_Human, Reactome_Pathways_2024, GO_Biological_Process_2023, all accessed via the Enrichr API endpoint [57]. **PROGENy and CollecTRI**: built-in resources of decoupler-py v2.1.6 [35,36,37]. **Cell–cell communication resource**: consensus LR pair set bundled with LIANA v1.7.1 [49,50]. **Drug repurposing libraries**: LINCS L1000 [55] via the Enrichr API; LINCS L1000 Chem Pert Consensus + up/down + Ligand Perturbations up/down [57]. **Drug–gene interactions and approval status**: DGIdb v5 [57] via the GraphQL endpoint at https://dgidb.org/api/graphql accessed on 12 May 2026. **Curated GMSC secretome v1.0**: provided as a supplementary CSV (data/curated/gmsc_secretome_v1.csv) with per-row DOI/PMID provenance and evidence-tier annotation. Code and reproducibility: All analysis code is deposited as a versioned repository at GitHub (https://github.com/SPatil555/oscc-gmsc-apoptosis-ros-pipeline accessed on 12 May 2026); a reviewer-only access link is available on request to the editor) and archived at Zenodo (10.5281/zenodo.20740146). The repository contains the two uv-managed Python lockfiles (envs/hybrid-main.lock, envs/hybrid-sc.lock), all analysis scripts, the pinned GDC release manifest, the versioned GMSC secretome CSV with provenance, and dated JSON caches of all LINCS and DGIdb API responses used in the manuscript.

## Supplementary Materials

The following supporting information can be downloaded at https://www.mdpi.com/article/10.3390/ijms27146480/s1.
