# Supplementary material for: An Integrative Bioinformatics Framework Prioritises a Gingival Mesenchymal Stem Cell Paracrine Apoptosis–ROS Axis in HPV-Negative Oral Squamous Cell Carcinoma: Preliminary Experimental Support and Repurposable-Drug Hypotheses
Source: Int J Mol Sci. 2026 Jul 21;27(14):6480. doi: 10.3390/ijms27146480 (PMC13411614; doi:10.3390/ijms27146480)
Supplement: Supplementary file 1 [file ijms-27-06480-s001.zip › ijms-4358044-supplementary figure _revision.pdf]

Supplementary figures

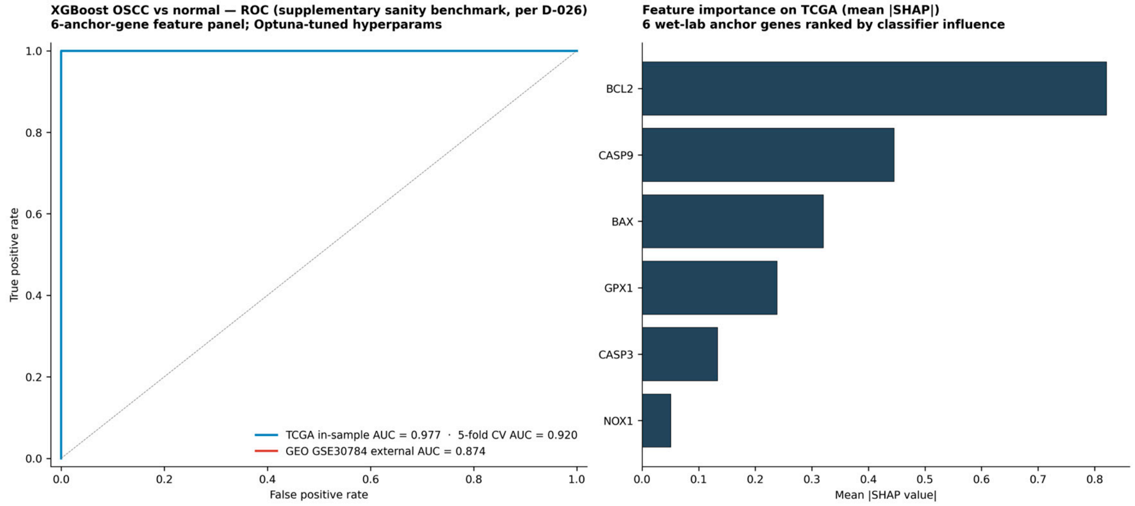

Figure S1. XGBoost OSCC vs. normal supplementary sanity classifier (per D-026; demoted from a headline result to a supplementary technical benchmark per Skeptic-S10 review). Left: ROC curves for TCGA-HNSC oral-cavity HPV-negative in-sample (AUC=0.977; 5-fold CV AUC=0.920) and external GEO GSE30784 (AUC=0.874). Right: SHAP mean-|value| feature importance over the six wet-lab anchor genes (ranked: BCL2, CASP9, BAX, GPX1, CASP3, NOX1). Reported as a technical sanity check rather than a biomarker claim.

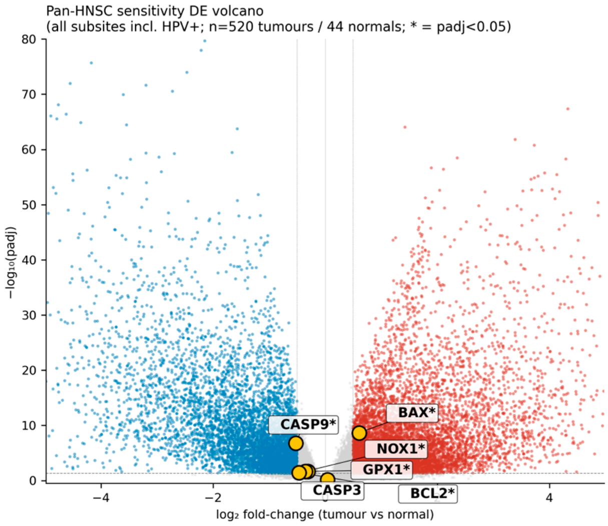

Figure S2. Pan-HNSC sensitivity differential-expression volcano including all anatomic subsites and HPV-positive tumours (n=520 tumours / 44 normals). At the larger sample size, all six wet-lab anchor genes (BAX, BCL2, CASP3, CASP9, NOX1, GPX1) reach significance at padj<0.05, a robustness check confirming that the strict HPV-negative stratification used in the main analysis tightens the contrast at the cost of some statistical power. Asterisks (*) mark padj<0.05.

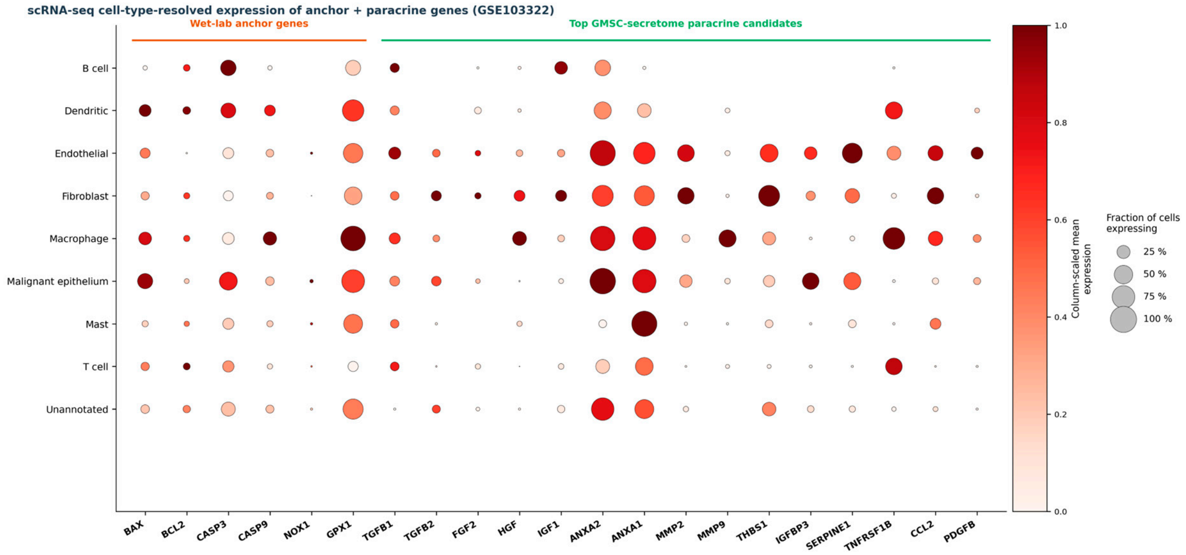

Figure S3. scRNA-seq cell-type-resolved expression matrix of the six wet-lab anchor genes plus the fifteen top GMSC-secretome paracrine candidates across the nine annotated cell-type clusters in GSE103322. Column-scaled mean expression (dot colour) and fraction of cells expressing (dot size); orange bracket = anchor genes; green bracket = GMSC-secretome paracrine candidates.

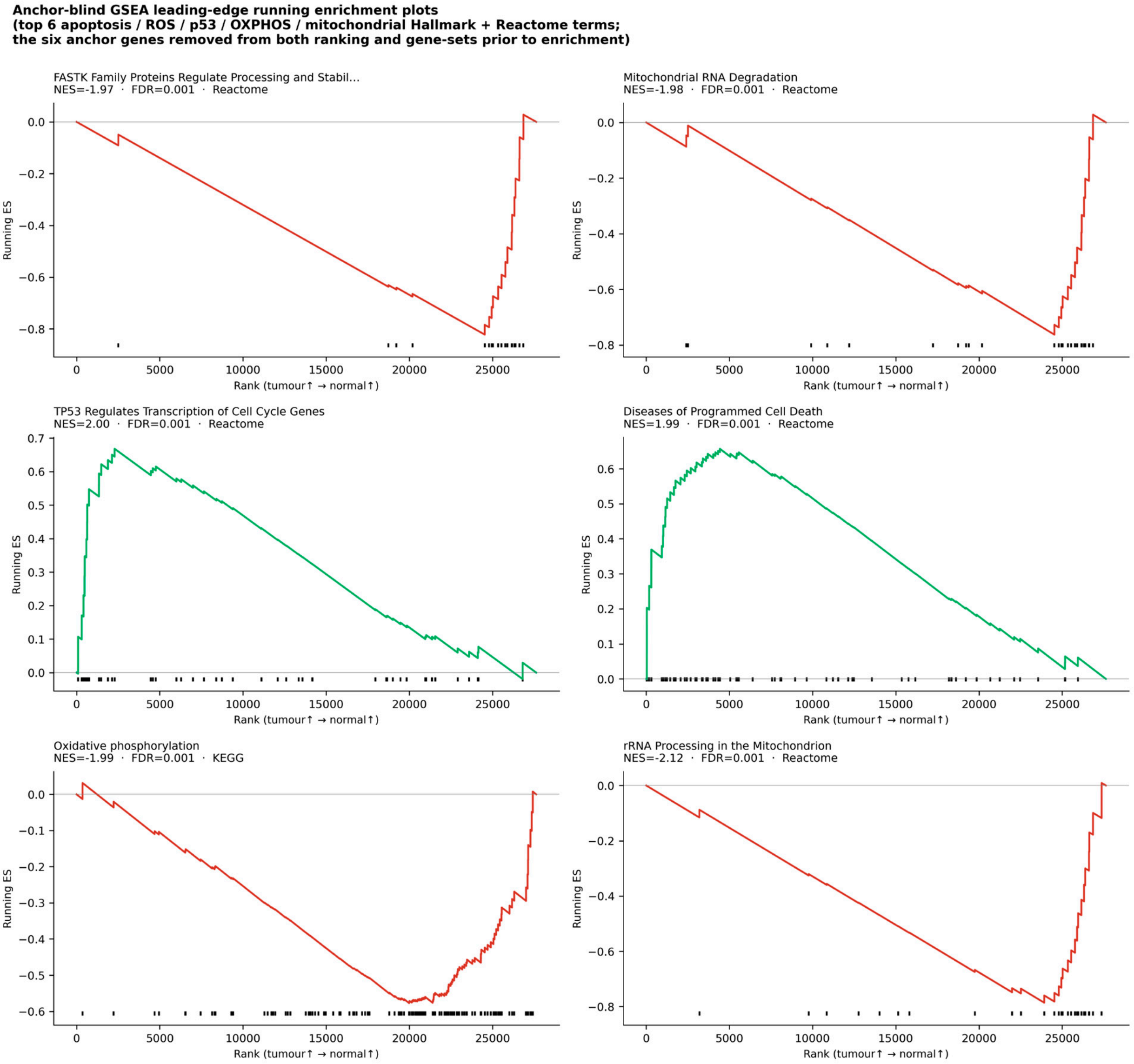

Figure S4. Blinded GSEA leading-edge running enrichment plots for the top six apoptosis / ROS / p53 / OXPHOS / mitochondrial Hallmark and Reactome terms (FASTK Family Proteins Regulate Mitochondrial RNA Processing; Mitochondrial RNA Degradation; TP53 Regulates Transcription of Cell Cycle Genes; Diseases of Programmed Cell Death; Hallmark Oxidative Phosphorylation; rRNA Processing in the Mitochondrion). Recovery is independent of the six wet-lab anchor genes, which were removed from both the ranked statistic and each gene-set's membership prior to enrichment computation.
