# Supplementary material for: An Integrative Bioinformatics Framework Prioritises a Gingival Mesenchymal Stem Cell Paracrine Apoptosis–ROS Axis in HPV-Negative Oral Squamous Cell Carcinoma: Preliminary Experimental Support and Repurposable-Drug Hypotheses
Source: Int J Mol Sci. 2026 Jul 21;27(14):6480. doi: 10.3390/ijms27146480 (PMC13411614; doi:10.3390/ijms27146480)
Supplement: Supplementary file 1 [file ijms-27-06480-s001.zip › ijms-4358044-supplementary tables.pdf]

Supplementary Tables S1–S11

Companion to: Gingival Mesenchymal Stem Cell Paracrine Signalling Targets an Apoptosis–ROS Axis in HPV-Negative Oral Squamous Cell Carcinoma: Patient-Cohort Validation and Repurposable Drug Candidates

Data sources: pinned GDC Data Release v45.0 (TCGA-HNSC); GEO accessions GSE30784, GSE25099, GSE37991, GSE103322; STRING v12.0; MSigDB v2023.1.Hs; LINCS L1000 via Enrichr; DGIdb v5 GraphQL. Numerical values exported from `results/tables/*.parquet* | .csv‘ produced by the analysis pipeline. The main Table 1 (qPCR primer sequences) appears in the main manuscript.

Supplementary Table S1. TCGA-HNSC cohort sample-size breakdown across HPV strata.

The HPV-negative oral-cavity column is the manuscript’s primary cohort. The anatomic-only column shows the larger but less biologically defensible cohort used as a sensitivity arm; it retains the 12 HPV-positive oral-cavity tumours that an ICD-10 filter alone cannot exclude.

**Sample group**	**HPV-negative oral cavity (primary)**	**Broad (HPV-negative or unknown)**	**Anatomic-only (HPV mixed)**	**Pan-HNSC (full TCGA-HNSC)**
Primary tumour (RNA-seq files)	**217**	233	245	520
Solid tissue normal	**15**	15	17	44
Metastatic	1	1	1	2
**OS event count** (deaths)	91	97	102	—
**Median follow-up time** (days)	—	—	—	—

Source: `results/tables/04_survival_sensitivity.csv‘ for cohort-level counts; HPV cross-reference from cBioPortal Datahub PanCanAtlas mirror (TCGA Network 2015 SUBTYPE field).

Supplementary Table S2. TCGA + GEO meta-DE consensus genes.

S2.A. Anchor-gene tumour versus normal differential expression across cohorts.

Gene	TCGA oral-cavity HPV− log_2FC	TCGA padj	TCGA-HNSC pan log_2FC	TCGA-HNSC pan padj	GEO meta log_2FC	GEO meta padj	I^2 (%)
**BAX**	+0.50	$1.84×10^{−2}$	+0.61	$2.21×10^{−9}$	+0.82	$6.29×10^{−5}$	88.1
**BCL2**	−1.28	$7.67×10^{−6}$	−0.47	$3.62×10^{−2}$	−0.61	0.18	92.4
**CASP3**	+0.24	0.13	+0.04	0.67	+0.13	0.64	93.0
**CASP9**	−0.54	$8.18×10^{−4}$	−0.52	$1.76×10^{−7}$	−0.55	$1.32×10^{−2}$	88.2
**NOX1**	−0.52	$6.01×10^{−2}$	−0.35	$2.23×10^{−2}$	−0.03	0.42	0
**GPX1**	−0.27	0.36	−0.30	$2.15×10^{−2}$	−0.15	0.52	90.5

S2.B. Top 20 GEO meta-DE genes (anchor-blind background).

Gene	log_2FC (meta)	p (meta)	padj (meta)	I^2 (%)	Concordant direction in TCGA?
MGP	−0.81	$1.1×10^{−16}$	$1.4×10^{−14}$	0	yes
PTGDS	−1.12	$1.1×10^{−16}$	$1.4×10^{−14}$	4.3	yes
CRAT	−0.78	$1.1×10^{−16}$	$1.4×10^{−14}$	0	yes
ZNF529	−0.46	$1.1×10^{−16}$	$1.4×10^{−14}$	5.2	yes
GULP1	−1.72	$1.1×10^{−16}$	$1.4×10^{−14}$	70.7	yes
TYRP1	−3.42	$1.1×10^{−16}$	$1.4×10^{−14}$	84.8	yes
POP7	+0.44	$2.2×10^{−16}$	$1.4×10^{−14}$	0	yes
ADAT1	+0.74	$2.2×10^{−16}$	$1.4×10^{−14}$	0	yes
ADCY6	−0.50	$2.2×10^{−16}$	$1.4×10^{−14}$	0	yes
CEP76	−0.63	$2.2×10^{−16}$	$1.4×10^{−14}$	11.6	yes
SLC15A3	+1.31	$2.2×10^{−16}$	$1.4×10^{−14}$	70.9	yes
CD97	+0.68	$2.2×10^{−16}$	$1.4×10^{−14}$	0	yes
NUPR1	−0.72	$2.2×10^{−16}$	$1.4×10^{−14}$	0	yes

NUTF2	+0.72	2.2×10^{-16}	1.4×10^{-14}	18.4	yes
OAS1	+0.75	2.2×10^{-16}	1.4×10^{-14}	0	yes
OAS3	+1.47	2.2×10^{-16}	1.4×10^{-14}	31.7	yes
GLB1	+0.61	2.2×10^{-16}	1.4×10^{-14}	46.9	yes
SLC6A4	−2.51	2.2×10^{-16}	1.4×10^{-14}	86.7	yes
ZNF585A	−0.60	2.2×10^{-16}	1.4×10^{-14}	0	yes
ADAM17	+0.45	2.2×10^{-16}	1.4×10^{-14}	37.7	yes

S2.C. Cross-cohort concordance summary.

**Metric**	**Value**
Genes in both TCGA + GEO meta	11,339
Co-significant (TCGA padj < 0.05 AND GEO padj_meta < 0.10)	2,470
Of those, sign-consistent direction	**2,431 (98.4%)**
Log_2 fold-change correlation (Pearson)	**r = 0.667 ($p < 10^{-300}$)**

Full per-gene tables: `results/tables/01_tcga_de_oc.csv‘ (28,669 genes), `results/tables/01_tcga_de_full_hnsc.csv‘ (32,217 genes), `results/tables/02_geo_meta_DE.csv‘ (12,736 genes), `results/tables/02_tcga_geo_concordance.parquet’.

Supplementary Table S3. Curated GMSC secretome v1.0.

Forty-seven gingival/MSC paracrine candidate factors with per-row DOI provenance and evidence-tier annotation. **Evidence tiers:** 1 = ≥2 GMSC-specific proteomics datasets; 2 = 1 GMSC-specific proteomics or strong narrative support in a canonical GMSC review; 3 = cross-MSC secretome evidence (BM-MSC, AD-MSC, DPSC); 4 = inferred from transcriptomics plus SignalP secretion annotation.

Gene	Protein name	Source DOI / PMID	Evidence tier	Source type
**TGFB1**	Transforming growth factor β1	10.3892/ijo.2016.3715 / PMID 27826624	2	proteomics CSM general
**TGFB2**	Transforming growth factor β2	10.3892/ijo.2016.3715 / PMID 27826624	3	proteomics CSM general
**TGFB3**	Transforming growth factor β3	10.3389/fimmu.2021.667221	3	review compendium
**IL6**	Interleukin 6	10.3389/fimmu.2021.667221	2	review compendium
**IL8**	Interleukin 8 / CXCL8	10.3389/fimmu.2021.667221	2	review compendium
**IL10**	Interleukin 10	10.3389/fimmu.2021.667221	2	review compendium
**IL1RN**	IL-1 receptor antagonist	10.3389/fimmu.2021.667221	3	review compendium
**VEGFA**	Vascular endothelial growth factor A	10.1016/j.jds.2024.06.002	2	review compendium
**HGF**	Hepatocyte growth factor	10.1016/j.jds.2024.06.002	2	review compendium
**FGF2**	Fibroblast growth factor 2	10.1016/j.jds.2024.06.002	2	review compendium
**IGF1**	Insulin-like growth factor 1	10.3389/fimmu.2021.667221	2	review compendium
**PDGFA**	Platelet-derived growth factor A	10.3389/fimmu.2021.667221	3	review compendium
**PDGFB**	Platelet-derived growth factor B	10.3389/fimmu.2021.667221	3	review compendium
**CXCL12**	C-X-C motif chemokine 12 (SDF-1)	10.3389/fimmu.2021.667221	2	review compendium
**CCL2**	C-C motif chemokine 2 (MCP-1)	10.3389/fimmu.2021.667221	3	review compendium
**CCL5**	C-C motif chemokine 5 (RANTES)	10.3389/fimmu.2021.667221	3	review compendium
**BMP2**	Bone morphogenetic protein 2	10.3389/fimmu.2021.667221	3	review compendium
**BDNF**	Brain-derived neurotrophic factor	10.3892/ijmm.2017.3231	3	proteomics CSM general
**NGF**	Nerve growth factor	10.3389/fimmu.2021.667221	3	review compendium
**GDNF**	Glial cell line-derived neurotrophic factor	10.3389/fimmu.2021.667221	3	review compendium
**MMP2**	Matrix metalloproteinase 2	10.3389/fimmu.2021.667221	2	review compendium

**MMP9**	Matrix metalloproteinase 9	10.3389/fimmu.2021.667221	2	review compendium
**TIMP1**	Tissue inhibitor of metalloproteinases 1	10.3389/fimmu.2021.667221	2	review compendium
**TIMP2**	Tissue inhibitor of metalloproteinases 2	10.3389/fimmu.2021.667221	2	review compendium
**THBS1**	Thrombospondin 1	10.3892/ijo.2016.3715	3	proteomics CSM general
**SPARC**	Secreted protein acidic and rich in cysteine	10.3892/ijo.2016.3715	3	proteomics CSM general
**COL1A1**	Collagen type I alpha 1	10.3892/ijo.2016.3715	3	proteomics CSM general
**COL3A1**	Collagen type III alpha 1	10.3892/ijo.2016.3715	3	proteomics CSM general
**FN1**	Fibronectin 1	10.3892/ijo.2016.3715	3	proteomics CSM general
**IGFBP3**	IGF binding protein 3	10.3389/fimmu.2021.667221	2	review compendium
**IGFBP5**	IGF binding protein 5	10.3389/fimmu.2021.667221	3	review compendium
**ANXA1**	Annexin A1	10.3892/ijmm.2017.3231	3	EV cargo GMSC
**ANXA2**	Annexin A2	10.3892/ijmm.2017.3231	3	EV cargo GMSC
**GAL1**	Galectin-1 (LGALS1)	10.3389/fimmu.2021.667221	3	review compendium
**FSTL1**	Follistatin-like 1	10.3389/fimmu.2021.667221	3	review compendium
**WNT5A**	Wnt family member 5A	10.3389/fimmu.2021.667221	3	review compendium
**DKK1**	Dickkopf-1	10.3389/fimmu.2021.667221	3	review compendium
**SERPINE1**	Plasminogen activator inhibitor-1 (PAI-1)	10.3389/fimmu.2021.667221	3	review compendium
**TNFRSF1B**	TNF receptor 1B (sTNFR2)	10.3389/fimmu.2021.667221	3	review compendium
**LIF**	Leukemia inhibitory factor	10.3389/fimmu.2021.667221	3	review compendium
**CD63**	CD63 tetraspanin	10.1186/s13287-020-00599-1	2	EV cargo GMSC
**CD81**	CD81 tetraspanin	10.1186/s13287-020-00599-1	2	EV cargo GMSC
**CD9**	CD9 tetraspanin	10.1186/s13287-020-00599-1	2	EV cargo GMSC
**HSP90AA1**	Heat shock protein 90α A1	10.1186/s13287-020-00599-1	3	EV cargo GMSC
**HSP70**	Heat shock protein 70 (HSPA1A)	10.1186/s13287-020-00599-1	3	EV cargo GMSC
**ALIX**	PDCD6IP	10.1186/s13287-020-00599-1	3	EV cargo GMSC

**TSG101**	Tumor susceptibility gene 101	10.1186/s13287-020-00599-1	3	EV cargo GMSC

Distribution: Tier 2 = 13 entries; tier 3 = 34 entries; and tier 1 = 0 entries (to be promoted from tier 2/3 as targeted GMSC proteomics is published in v1.1).

Machine-readable version with SHA256 hash and ISO extraction dates: `data/curated/gmsc_secretome_v1.csv‘ with provenance MANIFEST.

Supplementary Table S4. Anchor-blind PPI null-model output.

Top GMSC-secretome paracrine sources by count of significant source-to-target paths. Per the anchor-blind PPI shortest-path framework, the six wet-lab anchor genes are excluded from both source and target seed sets, and empirical p-values are computed against a 200-permutation degree-preserving null. Sources are ranked by count of significant source→target paths at empirical $p < 0.05$.

Source factor	Total reachable targets	Significant target paths ($p < 0.05$)	Median observed shortest-path length	Minimum length
**FGF2**	723	20	3	1
**TNFRSF1B**	718	17	3	1
**TGFB1**	722	16	2	0
**ANXA2**	723	15	3	1
**HGF**	722	14	3	0
**MMP9**	723	14	2	1
**TGFB2**	722	14	3	0
**IGF1**	723	14	2	0
**MMP2**	722	13	3	0
**CCL2**	722	13	3	1
**THBS1**	722	12	3	0
**IGFBP3**	722	12	3	0
**ANXA1**	722	12	3	0
**PDGFB**	721	11	3	1
**SERPINE1**	723	11	3	0

Pipeline parameters: STRING v12.0 high-confidence edges (combined_score ≥ 700; 231,568 edges); 200 degree-preserving rewires with 2× edge-count swap attempts each; per-pair empirical p = (null-count + 1) / (n_permutations + 1).

Quality gate (acceptance criterion #4): PASS — 42 of 47 secretome sources have ≥ 1 significant target path; 15 sources have ≥ 10 significant paths; total of 365 source–target pairs at empirical $p < 0.05$.

Full per-pair table: `results/tables/03_paracrine_predictions.csv‘ (30,285 rows).

S4.B. SIGNOR 3.0 + KEGG signalling overlay (pre-registered OmniPath fallback).

Because the OmniPath service backend was returning HTTP 502 at the time of analysis (documented in `data/reference/OMNIPATH_DEFERRED.md‘), the pre-registered fallback layer—SIGNOR 3.0 human relations plus KEGG signalling-pathway membership—was applied as the signed/directed annotation overlay on top of the STRING topology. SIGNOR adds curated cause/effect direction (activates, inhibits, complex) backed by published evidence; KEGG signalling membership flags whether a source–target pair sits in a canonical signal-transduction cascade.

Overlay metric	Value	Notes
Significant paracrine pairs (empirical $p < 0.05$)	**365**	from `03_paracrine_predictions.parquet’
With direct SIGNOR 3.0 evidence (either direction)	**1**	MMP9–TIMP1 (inhibits; PMID 36935521). SIGNOR is high-precision and most STRING shortest-path edges are not yet curated there
With shared KEGG signalling-pathway membership	**135 (37.0%)**	both endpoints in at least one common KEGG signalling pathway
Top KEGG signalling pathways among overlapping pairs	**MAPK (49), PI3K-Akt (42), Ras (29), TGF-β (23), AGE-RAGE (23), Rap1 (20), Calcium (19), Hippo (18), TNF (17), FoxO (10)**	canonical RTK / MAPK / PI3K-Akt / TGF-β cascades dominate, consistent with growth-factor / cytokine paracrine biology

Top mechanistically rich pairs (≥ 5 shared KEGG signalling pathways): IGF1→AKT2 (9 pathways), PDGFB→PDGFRB (7), PDGFB→PDGFA (7), TGFB3↔TGFB1/2 (5 each), PDGFA→PIK3CA (5), HGF→PDGFRB (5), FGF2→PDGFA (5), FGF2→HGF (5).

Interpretation: STRING + SIGNOR + KEGG-signalling functionally replaces OmniPath for the shortest-path use case. The 37% overlap with KEGG signalling membership is biologically meaningful—anchor-blind STRING shortest paths from GMSC-secretome ligands to the apoptosis × ROS module traverse the canonical RTK / MAPK / PI3K-Akt / TGF-β / Hippo / TNF signal-transduction routes, which is the expected paracrine-signalling biology. SIGNOR’s high-precision curation captures only the MMP9–TIMP1 inhibitory edge directly; the SIGNOR-only edge count is therefore a lower bound on signed evidence rather than a measure of biological coverage.

Source files: `results/tables/03_paracrine_signor_overlay.csv‘ (full per-pair annotation; 365 rows × 12 columns) and `results/tables/03_paracrine_signor_overlay_summary.json’.

Supplementary Table S5. Patient-cohort survival sensitivity across cohort variants × signature methods.

Multivariable Cox regression (covariates: age, sex) of three pre-registered signature variants applied to three cohort definitions. Log-rank p uses median-split stratification. LASSO-Cox rows that reach significance are highlighted.

Cohort	Method	n primary tumours	OS events	log-rank p	Cox HR (per z-unit)	95% CI lower	95% CI upper	Cox p
**HPV-negative oral cavity (primary cohort)**	z-sum (primary)	216	91	0.389	1.032	0.967	1.102	0.342
HPV-negative oral cavity	GSVA Hallmark Apop+ROS (sensitivity)	216	91	0.276	5.391	0.663	43.85	0.115
HPV-negative oral cavity	**LASSO-Cox Hallmark Apop+ROS (exploratory)**	216	91	**1.77×10^{-4}**	**301.2**	27.86	3258	**2.6×10^{-6}**
Broad HPV-negative or unknown oral cavity	z-sum	231	97	0.377	1.040	0.976	1.108	0.224
Broad HPV-negative or unknown oral cavity	GSVA Hallmark Apop+ROS	231	97	0.144	6.663	0.872	50.92	0.068
Broad HPV-negative or unknown oral cavity	**LASSO-Cox Hallmark Apop+ROS**	231	97	**5.76×10^{-5}**	**25.18**	7.20	88.12	**4.5×10^{-7}**
Anatomic-only (includes HPV+)	z-sum	243	102	0.213	1.048	0.984	1.116	0.146
Anatomic-only (includes HPV+)	GSVA Hallmark Apop+ROS	243	102	0.102	9.084	1.295	63.73	0.026
Anatomic-only (includes HPV+)	**LASSO-Cox Hallmark Apop+ROS**	243	102	**4.37×10^{-3}**	**427.2**	29.9	6104	**8.0×10^{-6}**

Interpretation: The pre-registered primary z-sum signature and pre-registered sensitivity GSVA score did not reach significance in the HPV-negative cohort. The pre-registered exploratory LASSO-Cox signature trained on the Hallmark Apoptosis + ROS feature pool (210 anchor-blind genes) stratifies overall survival across all three cohort variants (log-rank $p < 5 \times 10^{-5}$ in both strict and broad cohorts). The wide hazard-ratio confidence intervals reflect standardised-score units and small event counts; the *consistency* of the log-rank result is the stronger interpretive evidence.

Source: `results/tables/04_survival_sensitivity.csv'.

Supplementary Table S6. Pathway (PROGENy) and transcription-factor (CollecTRI) activity differences by z-sum stratum.

S6.A. PROGENy pathway activities (all 14 pathways).

Pathway	Median z-sum high	Median z-sum low	Δ (high−low)	Wilcoxon p	padj (FDR)
**Androgen**	6.52	6.85	−0.331	1.33×10^{-7}	1.86×10^{-6}
**VEGF**	3.77	3.44	**+0.323**	4.29×10^{-6}	3.0×10^{-5}
**TGFb**	11.3	12.2	**−0.887**	1.51×10^{-5}	**7.07×10^{-5}**
**TNFa**	5.84	5.49	+0.346	2.18×10^{-4}	7.63×10^{-4}
Oestrogen	−1.08	−0.92	−0.163	8.93×10^{-4}	2.50×10^{-3}
PI3K	−6.45	−6.21	−0.232	1.39×10^{-3}	3.25×10^{-3}
EGFR	−2.31	−2.47	+0.161	0.031	0.061
Hypoxia	5.93	6.20	−0.278	0.053	0.093
WNT	1.81	1.95	−0.139	0.061	0.095
MAPK	6.73	6.56	+0.168	0.20	0.28
NFkB	−0.45	−0.34	−0.106	0.28	0.36
p53	−0.50	−0.43	−0.072	0.34	0.40
JAK-STAT	14.8	14.8	−0.005	0.51	0.55
Trail	−3.66	−3.60	−0.056	0.83	0.83

Nine of 14 PROGENy pathways differential at FDR < 0.10.

S6.B. CollecTRI top 15 transcription-factor activities.

TF	Δ activity (high−low)	Wilcoxon p	padj (FDR)	Interpretation
**ZNF699**	−0.34	2.18×10^{-12}	1.64×10^{-9}	Down in pro-apoptotic stratum
**HOXA7**	−0.27	5.91×10^{-10}	1.51×10^{-7}	Down
**TCF12**	−0.52	6.70×10^{-10}	1.51×10^{-7}	Down (OSCC EMT-associated)
**POU3F2**	−0.26	8.04×10^{-10}	1.51×10^{-7}	Down
**SOX4**	−0.37	1.08×10^{-9}	1.63×10^{-7}	Down (OSCC stemness driver)

**NRG1**	−0.28	1.41×10^{-9}	1.77×10^{-7}	Down
**HDAC7**	+0.51	1.93×10^{-9}	1.86×10^{-7}	**Up**
**PRDM4**	−0.22	1.98×10^{-9}	1.86×10^{-7}	Down
**HDGF**	+0.33	2.81×10^{-9}	2.35×10^{-7}	**Up**
**SOX9**	−0.41	3.21×10^{-9}	2.42×10^{-7}	Down (OSCC invasion driver)
**RORA**	−0.33	4.55×10^{-9}	3.12×10^{-7}	Down
**CAMTA1**	+0.12	6.43×10^{-9}	4.04×10^{-7}	Up
**NCOA3**	−0.39	8.78×10^{-9}	4.65×10^{-7}	Down
**IKZF4**	−0.67	9.26×10^{-9}	4.65×10^{-7}	Down
**BCL11B**	−0.35	9.26×10^{-9}	4.65×10^{-7}	Down

A total of 461 of 753 CollecTRI TFs were differential at FDR < 0.10, with coordinated suppression of OSCC-progression factors (SOX4, SOX9, TCF12, POU3F2) and concomitant up-regulation of HDAC7/HDGF in the pro-apoptotic stratum.

Full tables: `results/tables/05_progeny_zsum_stratum_diff.csv‘ and `results/tables/06_tf_zsum_stratum_diff.csv’.

Supplementary Table S7. LIANA top CAF→malignant-epithelium ligand–receptor pairs.

Top 25 CAF→malignant-epithelium ligand–receptor pairs where the **ligand is in the curated GMSC secretome** (Supplementary Table S3). Ranked by LIANA consensus specificity_rank (geometric mean across CellPhoneDB + NATMI + Connectome). Receivers flagged for membership in Hallmark Apoptosis / ROS pathways are highlighted.

Ligand	Receptor	Specificity rank	Ligand ∈ GMSC secretome	Receptor in apoptosis/ROS module
**COL3A1**	MAG	$3.10{\times}10^{-4}$	✓	–
**IGF1**	**IGF1R**	$3.10{\times}10^{-4}$	✓	–
**CXCL12**	ITGA5	$3.10{\times}10^{-4}$	✓	–
**FN1**	**CD44**	$3.10{\times}10^{-4}$	✓	✓
**FN1**	ITGA2	$3.10{\times}10^{-4}$	✓	–
**FN1**	IL17RC	$3.10{\times}10^{-4}$	✓	–
**COL1A1**	ITGA5	$3.10{\times}10^{-4}$	✓	–
**THBS1**	SDC4	$3.10{\times}10^{-4}$	✓	–
**THBS1**	ITGA6	$3.10{\times}10^{-4}$	✓	–
**FN1**	SDC1	$3.10{\times}10^{-4}$	✓	–
**THBS1**	SDC1	$3.10{\times}10^{-4}$	✓	–
**TIMP2**	ITGA3	$3.10{\times}10^{-4}$	✓	–
**FN1**	ITGA5_ITGB1	$3.10{\times}10^{-4}$	✓	–
**FN1**	ITGA6	$3.10{\times}10^{-4}$	✓	–
**TIMP2**	**CD44**	$3.10{\times}10^{-4}$	✓	✓
**IGF1**	**IGF2R**	$3.10{\times}10^{-4}$	✓	✓
**IGF1**	ITGA6_ITGB4	$3.10{\times}10^{-4}$	✓	–
**CCL2**	CCR3	$3.15{\times}10^{-4}$	✓	–
**FN1**	MAG	$3.68{\times}10^{-4}$	✓	–
**TGFB3**	**ACVR1_TGFBR1_TGFBR2**	$4.27{\times}10^{-4}$	✓	–

**TGFB3**	ACVR1B_TGFBR2	$4.27{\times}10^{-4}$	✓	—
**TGFB3**	ACVR1_TGFBR2	$4.27{\times}10^{-4}$	✓	—
**TGFB3**	ACVR1C_TGFBR2	$4.27{\times}10^{-4}$	✓	—
**TGFB3**	TGFBR1_TGFBR2	$4.27{\times}10^{-4}$	✓	—
**TGFB3**	ITGB6	$4.75{\times}10^{-4}$	✓	—

A total of 4,012 CAF→malignant LR pairs were identified by LIANA consensus; full table in `results/tables/08_liana_lr_pairs.parquet'. Cross-referenced with the curated GMSC secretome (Supplementary Table S3) to identify GMSC-paracrine-plausible axes.

Supplementary Table S8. Anchor-blind GSEA top apoptosis / ROS / p53 / OXPHOS / mitochondrial hits.

Top 15 anchor-blind GSEA hits relevant to the apoptosis × ROS programme. The six wet-lab anchor genes were excluded from both the ranking statistic and each gene-set's membership prior to enrichment computation.

Library	Term	NES	FDR q-value
Reactome Pathways 2024	FASTK Family Proteins Regulate Processing and Stability of Mitochondrial RNAs	−1.97	0.001
Reactome Pathways 2024	Mitochondrial RNA Degradation	−1.98	0.001
**Reactome Pathways 2024**	**TP53 Regulates Transcription of Cell Cycle Genes**	**+2.00**	**0.001**
**Reactome Pathways 2024**	**Diseases of Programmed Cell Death**	**+1.99**	**0.001**
KEGG 2021 Human	Oxidative phosphorylation	−1.99	0.001
Reactome Pathways 2024	rRNA Processing in the Mitochondrion	−2.12	0.001
Reactome Pathways 2024	tRNA Processing in the Mitochondrion	−2.23	0.001
Reactome Pathways 2024	Mitochondrial Protein Degradation	−2.20	0.001
Reactome Pathways 2024	TP53 Regulates Transcription of Genes Involved in G2 Cell Cycle Arrest	+1.92	0.001
Reactome Pathways 2024	Regulation of TP53 Activity Through Phosphorylation	+1.94	0.001
**MSigDB Hallmark 2020**	**Oxidative Phosphorylation**	**−2.33**	**0.001**
Reactome Pathways 2024	Mitochondrial Fatty Acid Beta-Oxidation	−1.93	0.00121
Reactome Pathways 2024	TP53 Regulates Transcription of Genes Involved in G1 Cell Cycle Arrest	+1.86	0.00155
GO Biological Process 2023	Oxidative Phosphorylation (GO:0006119)	−1.97	0.00161
GO Biological Process 2023	Mitochondrial ATP Synthesis Coupled Electron Transport (GO:0042775)	−1.97	0.00202

Across all four libraries: 744 hits at FDR < 0.05; 1,013 hits at FDR < 0.10. Because the anchor genes were removed from both the ranking and gene-set definitions before enrichment was computed, the apoptosis/ROS hallmark recovery is not statistically tautological and reflects independent transcriptomic context.

Full GSEA output: `results/tables/07_gsea_anchor_blind.csv‘ (7,650 rows).

Supplementary Table S9. Immune microenvironment by z-sum stratum.

S9.A. Cell-type activity differences (decoupler ULM on 17-cell-type CellMarker 2.0 panel).

Cell type	Median z-sum high	Median z-sum low	Δ (high − low)	Wilcoxon p	padj (FDR)
**Endothelial**	2.42	2.72	−0.31	9.95×10^{-7}	**1.49×10^{-5}**
**Fibroblast/CAF**	3.76	4.22	−0.46	6.64×10^{-6}	**4.98×10^{-5}**
**Mast cell**	−0.07	0.45	−0.52	4.67×10^{-4}	**2.34×10^{-3}**
**Monocyte**	3.68	3.84	−0.15	3.04×10^{-3}	**1.14×10^{-2}**
**B cell**	−0.86	−0.57	−0.29	1.35×10^{-2}	**3.82×10^{-2}**
**Dendritic conventional**	−0.10	+0.05	−0.15	1.53×10^{-2}	**3.82×10^{-2}**
**Macrophage M2**	1.53	1.70	−0.17	2.12×10^{-2}	**4.53×10^{-2}**
T cell regulatory	0.78	0.99	−0.22	3.10×10^{-2}	5.81×10^{-2}
Plasma B cell	2.02	2.47	−0.45	8.82×10^{-2}	0.147
Macrophage M1	0.58	0.50	**+0.08**	0.22	0.33
T cell (total)	1.04	1.24	−0.20	0.33	0.45
Neutrophil	0.22	0.34	−0.12	0.43	0.53
T cell CD8	0.42	0.49	−0.07	0.53	0.61
T cell exhausted	0.34	0.46	−0.12	0.57	0.61
NK cell	0.06	0.13	−0.07	0.95	0.95

Interpretation: The pro-apoptotic stratum is a **stromal-cool, lower-checkpoint** phenotype—reduced endothelial, fibroblast/CAF, mast, monocyte, B cell, dendritic and macrophage M2 activities, with macrophage M1 trending up (the only cell type with a positive delta).

S9.B. Immune-checkpoint marker expression (VST log-units).

Gene	Median z-sum high	Median z-sum low	Δ	Wilcoxon p	padj (FDR)
**BTLA**	4.23	4.75	**−0.52**	2.46×10^{-4}	**2.21×10^{-3}**
ICOS	6.87	7.21	−0.34	0.039	0.174
TIGIT	7.77	8.20	−0.43	0.088	0.264

LAG3	8.36	8.03	+0.32	0.319	0.718
CTLA4	7.24	7.56	−0.32	0.490	0.883
VTCN1 (B7-H4)	5.37	5.29	+0.08	0.778	0.913
HAVCR2 (TIM-3)	8.90	8.84	+0.06	0.796	0.913
CD274 (PD-L1)	8.92	9.08	−0.16	0.885	0.913
PDCD1 (PD-1)	6.25	6.44	−0.19	0.913	0.913

Only BTLA reaches statistical significance after FDR adjustment; PD-L1, PD-1, CTLA4, TIGIT and ICOS all trend lower in the pro-apoptotic stratum without reaching FDR < 0.10 individually.

Sources: `results/tables/10_immune_zsum_stratum_diff.csv‘, `results/tables/10_immune_checkpoint_expression.csv’.

Supplementary Table S10. LINCS L1000 drug repurposing—top compounds with DGIdb v5 annotation.

Top 28 compounds from anchor-blind LINCS L1000 enrichment, cross-annotated with DGIdb v5 (drug–target interactions, FDA approval status, drug class). The FDA-approved compound mitoxantrone is highlighted.

Compound	Library hits	Best FDR	FDA approved	Top DGIdb targets	Drug class
**NVP-AUY922** (luminespib)	3	0.247	No	HSP90AA1, HSP90AA2P, HSP90AB1	HSP90 inhibitor / antineoplastic
**ZSTK-474**	3	0.247	No	PIK3CA/CB/CD/CG, PIK3R1/2/3/5	PI3K inhibitor (pan-class)
**TGX-221**	3	0.300	No	BRAF, F7, JAK2, PIK3CA, PIK3CB, PTEN	PI3K / multi-kinase
**BMS-345541**	3	0.426	No	EGFR, ERBB2, ERBB4	Kinase inhibitor
**GDC-0879**	3	0.426	No	(annotated)	BRAF inhibitor
**GSK-1070916**	3	0.426	No	BCL2, NR1D1, NR1H3	Aurora kinase inhibitor
**PIK-93**	3	0.426	No	F5, F7, PIK3CB	PI3K class IA inhibitor
**AZD-5438**	3	0.521	No	CHRM3	CDK inhibitor (CDK1/2/9)
**AZD-6482**	3	0.521	No	(annotated)	PI3Kβ inhibitor
**AZD-7762**	3	0.521	No	(annotated)	CHK1 inhibitor
**AZD-8055**	3	0.521	No	(annotated)	mTOR kinase inhibitor
**AZD-8330**	3	0.521	No	(annotated)	MEK1/2 inhibitor
**BIX-01294**	3	0.521	No	ATAD5, ATXN2, FEN1, GCNT2, IDH1, MTOR, OPN1MW, SMAD3	G9a/EHMT2 methyltransferase inhibitor
**GDC-0068**	3	0.521	No	(annotated)	AKT1/2/3 inhibitor
**GDC-0980**	3	0.521	No	(annotated)	PI3K/mTOR inhibitor
**GSK-2126458**	3	0.521	No	BCL2, NR1D1, NR1H3	PI3K/mTOR inhibitor
**GSK-690693**	3	0.521	No	BCL2, NR1D1, NR1H3	AKT inhibitor
**NVP-AEW541**	3	(annotated)	No	BRAF, DPH1, PIK3CA, PTEN	IGF1R inhibitor
**NVP-TAE684**	3	(annotated)	No	ABL1, ACVR1, AURKA, AURKB, AXL, BLK, BRSK1, BTK, CAMK1D, CAMK2A	ALK / multi-kinase inhibitor (103 interactions)
**OSI-027**	3	(annotated)	No	RICTOR, RPTOR	mTORC1/2 inhibitor

**PHA-665752**	3	0.300	No	TP53	c-MET inhibitor
**PHA-767491**	3	(annotated)	No	TP53	CDC7 / CDK9 inhibitor
**PLX-4720**	3	(annotated)	No	CSF1R, NTRK1, NTRK2, NTRK3	BRAF V600E inhibitor (vemurafenib precursor)
**saracatinib**	3	(annotated)	No	ABL1, ALK, BCR, BRAF, DDR2, EML4, EPHX2, FKBP1AP3, IDH1, MS4A2	SRC family inhibitor
**dinaciclib**	3	(annotated)	No	ALK, CCNE1, CDK1, CDK2, CDK5, CDK9, EGR4, EIF4A1, EIF4E, EIF4G1	Pan-CDK inhibitor
**mitoxantrone**	3	(annotated)	**YES (1987)**	**ABCB1, ABCG2, ARSA, ATAD5, ATXN2, BAX, BCL2, BLM, BRCA1, C10orf67**	**Antineoplastic; small molecule; TOP2 inhibitor**
**geldanamycin**	3	(annotated)	No	ATAD5, CD28, CDC37, CEL, EGFR, FCGR3A, FCGR3B, FLT3, HIF1A, HSP90AA1	HSP90 inhibitor / antineoplastic
**radicicol**	3	(annotated)	No	HSP90AA1, HSP90AB1, NTRK1	HSP90 inhibitor / antineoplastic

Key finding: Mitoxantrone is the only FDA-approved compound among the top 150 reverse-signature hits, with 44 curated DGIdb interactions including **direct binding to BAX and BCL2**—among the most direct mechanistic bridges from the repurposing screen to the wet-lab anchor genes.

HSP90 inhibitor class converges across NVP-AUY922 (luminespib; clinically tested in NSCLC), geldanamycin (prototype HSP90 inhibitor) and radicicol—all DGIdb-annotated as antineoplastic with HSP90 family targets.

Source: `results/tables/09_drug_repurposing_annotated.csv‘ and dated DGIdb cache JSON in `data/lincs/cache/’.

Supplementary Table S11. XGBoost supplementary sanity classifier (OSCC-vs-normal).

Per D-026 (acceptance criterion #7 removed): This analysis is a **technical sanity benchmark, not a biomarker claim**.

S11.A. Classifier performance metrics.

Cohort	n total	n tumour	AUC	PR-AUC	5-fold CV AUC
TCGA-HNSC oral-cavity HPV-neg (in-sample)	262	245	0.977	0.998	0.920
GEO GSE30784 (external validation, after platform alignment)	212	167	0.874	0.969	—

S11.B. SHAP feature importance over the six anchor genes.

Anchor gene	Mean |SHAP| value	Direction in classifier
**BCL2**	0.821	dominant—lower in tumour (anti-apoptotic loss)
**CASP9**	0.445	next-most important—lower in tumour
**BAX**	0.320	higher in tumour (pro-apoptotic increase)
**GPX1**	0.239	lower in tumour
**CASP3**	0.134	minor contribution
**NOX1**	0.050	smallest contribution

Best Optuna hyperparameters (30-trial Bayesian search, 5-fold stratified CV): `n_estimators=150, max_depth=2, learning_rate=0.013, subsample=0.93, colsample_bytree=0.72, reg_alpha=0.148, reg_lambda=0.014, min_child_weight=2'.

Source: `results/tables/11_classifier_metrics.parquet' and `results/tables/11_classifier_shap.csv'.
